# Circuit-level theories for sensory dysfunction in autism: convergence across mouse models

**DOI:** 10.3389/fneur.2023.1254297

**Published:** 2023-09-07

**Authors:** Hannah R. Monday, Han Chin Wang, Daniel E. Feldman

**Affiliations:** Department of Molecular and Cell Biology, Helen Wills Neuroscience Institute, University of California, Berkeley, Berkeley, CA, United States

**Keywords:** autism, sensory, cortex, theory, excitability, neural coding, circuit, inhibition

## Abstract

Individuals with autism spectrum disorder (ASD) exhibit a diverse range of behavioral features and genetic backgrounds, but whether different genetic forms of autism involve convergent pathophysiology of brain function is unknown. Here, we analyze evidence for convergent deficits in neural circuit function across multiple transgenic mouse models of ASD. We focus on sensory areas of neocortex, where circuit differences may underlie atypical sensory processing, a central feature of autism. Many distinct circuit-level theories for ASD have been proposed, including increased excitation–inhibition (E–I) ratio and hyperexcitability, hypofunction of parvalbumin (PV) interneuron circuits, impaired homeostatic plasticity, degraded sensory coding, and others. We review these theories and assess the degree of convergence across ASD mouse models for each. Behaviorally, our analysis reveals that innate sensory detection behavior is heightened and sensory discrimination behavior is impaired across many ASD models. Neurophysiologically, PV hypofunction and increased E–I ratio are prevalent but only rarely generate hyperexcitability and excess spiking. Instead, sensory tuning and other aspects of neural coding are commonly degraded and may explain impaired discrimination behavior. Two distinct phenotypic clusters with opposing neural circuit signatures are evident across mouse models. Such clustering could suggest physiological subtypes of autism, which may facilitate the development of tailored therapeutic approaches.

## Introduction

Autism spectrum disorder (ASD) is a neurodevelopmental disorder characterized by impaired social communication, restricted or repetitive behaviors or interests, and atypical sensory processing. ASD is extremely diverse, with widespread phenotypic differences among individuals and hundreds of risk genes (1). These ASD genes do not share a single common molecular or cellular function, though they tend to be involved in chromatin remodeling, mRNA translation, and synapse function (2). A major goal in autism research has been to test whether distinct gene mutations drive convergent pathophysiology at the level of molecular pathways, synapse function, neural circuits, or neural coding, which may underlie the shared core features of the condition. Whether such convergence exists, and at what level, remains unknown. In principle, distinct gene mutations could converge on a single shared neurobiological impairment (indicating a common basis for ASD), a small set of distinct impairments (indicating different functional clusters of ASD), or no common impairment (indicating that ASD is actually a large constellation of pathophysiologically distinct conditions).

Here, we review theories of autism at the neural circuit and neural coding levels and evaluate evidence for convergence across different genetic forms of autism. We focus on the primary sensory areas of the cerebral cortex because these have been intensively studied and because atypical sensory processing is a common feature of people with ASD and has been a diagnostic criterion since DSM-5 (3). Cellular, synapse, and circuit dysfunction have been extensively studied in the primary somatosensory (S1), visual (V1), and auditory (A1) cortex in many transgenic mouse models of ASD. This review focuses on these three cortical areas because the large number of studies in different ASD models provides a strong test case for pathophysiological convergence. Circuit dysfunction in these areas may underlie atypical sensory processing in autism. Multisensory, social, and motor phenotypes in ASD likely reflect dysfunction in other cortical areas (4, 5), but may be caused by similar cellular and circuit deficits as in the sensory cortex because of shared circuit architecture and developmental principles across the neocortex.

### Why focus on the sensory cortex? Sensory processing impairments in ASD

Up to 90% of people with ASD show atypical sensory processing, which is often classified into hyper-responsiveness, hypo-responsiveness, sensory avoidance, and sensory-seeking behaviors (3, 6). These behavioral symptoms suggest dysfunction in sensory brain regions, which is often observed in brain-based measurements (4).

Behaviorally, multiple sensory domains are often affected (6). Hypo-responsiveness and hyper-responsiveness can occur in the same individuals (7). Hypo-responsiveness is as pronounced as hyper-responsiveness across clinical studies (8) and in first-person accounts (9). Sensory seeking is thought to reflect underlying hyposensitivity to sensory stimuli, while sensory avoidance may be driven by hypersensitivity. Individual syndromic forms of autism also exhibit a mixture of these sensory phenotypes across different sensory modalities, depending on the type of stimulus and its social relevance (10, 11). Cluster analyses of sensory profiles of individuals with autism tend to reveal two main behavioral subgroups: one that is impaired relative to typically developing individuals across all four sensory domains (poor registration, sensitivity, sensory seeking, and sensory avoiding), and one that shows no impairment across any of the four (12–14). Recent work suggests smaller clusters of individuals with deficits in particular sensory modalities exist (15).

More specific psychophysical characterization of processing impairments is rare and usually performed in high-functioning individuals. Some of the most common findings from psychophysical studies in touch, vision, and hearing are lower tactile thresholds for detection of vibration and painful stimuli (7, 16), auditory hyper-responsiveness (17–20), difficulty in distinguishing speech in noise (21, 22), impaired temporal processing of sounds (23), elevated visual detail detection (24), weak binocular rivalry, altered visual motion processing (25), and possibly face perception deficits (26). These sensory impairments are reviewed in detail elsewhere (4, 5, 18, 27). Psychophysical differences across more severe, syndromic forms of autism are generally less known. In the sensory cortex, functional magnetic resonance imaging (fMRI) and electroencephalography (EEG) studies have identified varied phenotypes in people with ASD, including hypo- and hyperactivation by sensory stimuli. fMRI findings include hyperactivation by visual and auditory stimuli, which may relate to behavioral hypersensitivity (28), abnormal somatosensory maps, and decreased habituation in the somatosensory cortex to mildly aversive tactile stimuli (29). This suggests that the sensory cortex is a site of processing abnormalities in autism, a view that is supported by a recent study that found that, of all brain areas, primary sensory cortices show the most profound gene expression changes in people with ASD (30).

“Sensory-first” theories of autism posit that impairments in early sensory processing (including in the primary sensory cortex) may lead to widespread effects that contribute to impairments outside of the sensory domain. For example, touch and tactile perception are essential during infancy for the development of secure attachments with caregivers and continue to support social functioning throughout life (31). Impairments in somatosensation leading to tactile aversion could thereby contribute to social dysfunction and anxiety in individuals with ASD, as suggested by recent studies in mice (32). Similarly, impairments in early visual processing or in the processing of face information could drive reduced eye contact, which is an early indicator of autism, persists through adulthood, and may be relevant for social impairments in autism (4). Moreover, the mechanism of circuit disruption in sensory areas may occur commonly throughout the cortex and underlie both sensory and non-sensory features of ASD.

### Genetic mouse models of ASD provide a basis for studying convergence

Nearly all widely used transgenic mouse models of ASD model severe monogenic, syndromic forms of autism, caused by loss of function in a single gene. These include fragile X syndrome (FXS, caused by loss of function in the *FMR1* gene), Rett Syndrome (loss of function in the *MECP2* gene), Tuberous Sclerosis Complex (loss of function in either the *TSC1* or *TSC2* gene), Phelan-McDermid syndrome (PMS, loss of function in the *SHANK1, 2*, or *3* genes), and Angelman syndrome (loss of function in the *UBE3A* gene). These mice exhibit abnormal social interactions and repetitive behavior, as well as sensory processing impairments reminiscent of human autism. In humans, many of these syndromes are also associated with intellectual disability, cognitive impairment, and/or seizures, and these mouse models often exhibit corresponding behavioral phenotypes. Other transgenic rare syndromic ASD models in which the sensory cortex has been substantially studied include *Cntnap2–/–, Syngap1*+*/–, Scn1a*+*/–, Scn2a*+*/–*, and *Arid1b*+*/–* mice.

These ASD models exhibit a range of sensory behavioral abnormalities, which may reflect atypical sensory processing in autism (31). In the tactile domain, many ASD models (including *Mecp2* null, *Shank3B–/–, Fmr1–/–, Gabrb3*+*/–*, and *Syngap1*+*/–* mice) show impaired recognition of novel textured objects, interpreted as a deficit in texture discrimination (32–35). *Syngap1*+*/–* mice also show impaired tactile sensory detection on an operant task (34). In contrast, *Shank3B–/–* mice show enhanced detection of weak tactile stimuli (36), and *Fmr1* mice show impaired adaptation that causes innate tactile defensiveness (37). Innate sensitivity to touch and painful stimuli is enhanced in *Fmr1–/–, Shank3B–/–, Mecp2 null, Gabrb3*+*/–*, and *Cntnap2–/–* mice (32, 35, 38), while pain sensitivity is decreased in *Shank2–/–* mice (39). In the visual domain, orientation discrimination is impaired in *Fmr1–/–* mice (40); visual contrast detection is impaired in *Cntnap2–/–* mice (41); and visual form discrimination learning is slowed in *Ube3a m–/p*+ mice (42). In contrast, a variety of innate visual detection behaviors are enhanced, including the optokinetic reflex in *Mecp2* duplication mice (43), and visually evoked fidget responses in *Fmr1–/–* mice (44). Visual acuity is also enhanced in *Mecp2* duplication mice (45). In the auditory domain, *Fmr1–/–* and *Cntnap2–/–* mice show increased acoustic startle (19, 46) and audiogenic seizures (47), and *Fmr1–/–* mice show normal tone detection performance on an operant task, but reduced reaction times that suggest increased perceived loudness (48).

This diversity of sensory deficits parallels the broad range of sensory phenotypes in people with ASD, but is there any convergence in sensory deficits across different mouse models? While behavioral task design varies across studies, a strong trend is apparent in the evidence. Across mouse models, innate sensory detection-related behaviors are reliably elevated (e.g., paw withdrawal to a touch stimulus, acoustic startle, and visually evoked fidget behavior), operantly trained detection behaviors show a mix of enhancement and impairment (e.g., operant visual or whisker touch detection), and innate or operant sensory discrimination behaviors are reduced or impaired (e.g., textured novel object recognition and operantly trained visual discrimination). This suggests that subcortical sensory processing (subserving innate detection) may be hypersensitive across many models, whereas cortical processing (mediating more complex discrimination and some operantly learned detection behaviors) may be degraded.

Using these mouse models, many studies have measured molecular, cellular, and circuit correlates of ASD in the sensory cortex. These provide the main data for evaluating possible pathophysiological convergence in brain function across genetic forms of ASD. Mouse models also provide a platform for testing potential therapeutic approaches to normalize cellular and circuit function and behavior. However, it is important to recognize that these mice all model severe syndromic forms of autism that are only a small fraction of autism cases in humans and do not include milder, higher-functioning forms of autism. Lessons from existing mouse models are therefore unlikely to be relevant across the entire autism spectrum.

## Theories of sensory circuit dysfunction in ASD

**Figure 1:**
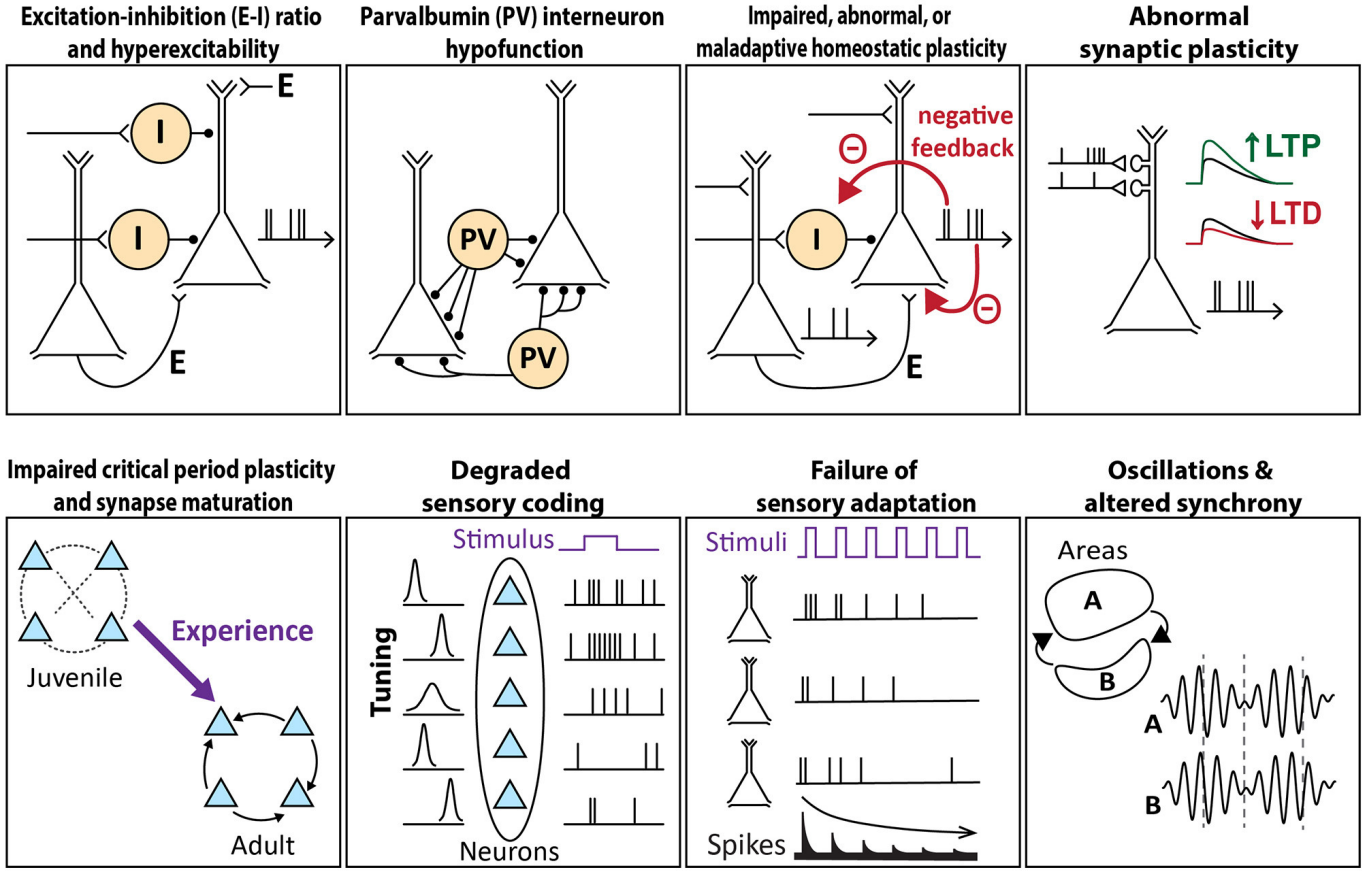
Schema of circuit mechanisms underlying sensory processing that are theorized to go awry in autism. Each image illustrates the normal circuit function, a breakdown of which may contribute to sensory features of autism.

This section will describe and evaluate common mechanistic theories of cortical circuit dysfunction in ASD (schematized in Figure 1). This overview will position us to evaluate, for each theory, the evidence for convergence across various genetic mouse models of autism.

### Excitation–inhibition (E–I) ratio and hyperexcitability

One of the first cellular-circuit theories of ASD was the E–I ratio hypothesis. This hypothesis proposes that in ASD, cortical circuits exhibit reduced synaptic inhibition or increased synaptic excitation onto pyramidal (PYR) cells, and that the resulting increase in E–I ratio drives circuit hyperexcitability and excess PYR spiking (49, 50). In support of this idea, a reduction of GABAergic markers is commonly observed in the brains of people with autism, which could potentially drive hyperexcitability, and may explain why autism is associated with epilepsy in 30% of individuals (51). In the sensory cortex, circuit hyperexcitability could predict sensory hypersensitivity in autism (52). But atypical sensory processing in autism is not just hypersensitivity, and indeed, hyposensitivity and sensory seeking are common, so circuit hyperexcitability is unlikely to provide a full explanation. Moreover, the diversity of inhibitory cell types and circuits in the cortex complicates the idea of a simple “E–I ratio,” and it is now understood that transient imbalances and delays between E and I are a critical aspect of normal information processing in the cortex. While inhibition is often reduced in the ASD mouse cortex, this only in some cases leads to consequential changes in PYR spiking (53). Therefore, the E–I ratio and hyperexcitability hypothesis does not appear to account for sensory cortex dysfunction in the majority of autism mouse models.

### Parvalbumin (PV) interneuron hypofunction

The parvalbumin (PV) hypothesis of ASD posits that cortical dysfunction originates from the hypofunction of PV-positive interneuron circuits (54). PV interneurons represent ~40% of cortical interneurons and provide powerful perisomatic inhibition to PYR cells (55). PV cells have critical information processing functions in the sensory cortex, including gain modulation, sharpening sensory tuning, generating gamma rhythms, enforcing precise spike timing, and modulating noise correlations. In post-mortem brains of people with ASD, PV is the most downregulated mRNA transcript, and the number of PV-positive neurons is diminished (56, 57). In studies of ASD mouse models, reduced PV cell number, reduced PV protein expression, or impaired PV circuit function are commonly featured (58, 59). PV cell and circuit hypofunction could detrimentally elevate the PYR firing rate, and even if the PYR firing rate remains normal, it could muddy neural codes by broadening tuning, impairing gain regulation, disturbing gamma synchronization, or degrading spike timing. PV neurons also regulate critical period plasticity, and thus PV hypofunction could alter or impair activity-dependent circuit development, producing abnormal or persistently immature circuits (60). Ample evidence exists for PV hypofunction in ASD, which could drive many of the sensory phenotypes. PV function may be altered directly by some ASD gene mutations. Alternatively, because PV circuits are highly plastic as part of the brain's endogenous homeostatic mechanisms, PV hypofunction could arise as a compensatory mechanism that is recruited in response to abnormal cortical activity (61).

### Impaired, abnormal, or maladaptive homeostatic plasticity

Homeostatic plasticity is a negative-feedback process that dynamically adjusts excitation, inhibition, and/or intrinsic excitability to stabilize network activity. Impaired or abnormal homeostatic plasticity has been proposed to drive atypical firing rates or patterns, disrupting the flow of information in cortical circuits and driving sensory processing impairment in ASD (62). Multiple homeostatic mechanisms exist in cortical circuits, and function together to actively maintain the PYR cell firing rate near a setpoint value that may represent the optimal firing rate for encoding information (63). These mechanisms include synaptic scaling, which multiplicatively adjusts excitatory or inhibitory synaptic weights on single PYR cells; homeostatic plasticity of PYR cell intrinsic excitability; and PV circuit plasticity, in which the gain of PV interneuron circuits is adjusted in response to mean activity in local networks. These processes work at different time scales, but all have the net effect of preventing the mean firing rates of local PYR cells from deviating from their setpoint.

If homeostatic plasticity is impaired or abnormal in ASD, networks would lose the ability to maintain stable PYR firing rates. In support of this theory, ASD risk genes span a range of cellular processes, including activity-dependent transcription, translation, energy metabolism, synaptic function, and intrinsic excitability, that are expected to contribute to homeostatic plasticity (64). This suggests that ASD-linked mutations may abolish or dysregulate key forms of neuronal homeostasis, so that PYR activity is destabilized or abnormal (62). Alternatively, mutations may alter homeostatic plasticity to make it maladaptive, in the sense that it could succeed in stabilizing firing rates, but at the cost of degrading some other critical aspect of neural coding (53). Several genes associated with syndromic autism have been linked to the failure of homeostatic mechanisms, suggesting that impaired or abnormal homeostasis could be a convergent explanation for multiple forms of ASD in humans (65). In this view, the diverse array of synaptic and intrinsic physiological abnormalities observed across different ASD mouse models may reflect the failure of homeostasis to reset normal values for these parameters.

### Abnormal synaptic plasticity

Impairments in long-term synaptic plasticity, including long-term potentiation (LTP) and long-term depression (LTD) at excitatory synapses onto PYR cells, are prevalent across mouse models of autism. These impairments may not only impact learning and memory but also drive sensory dysfunction by impairing the use-dependent refinement of circuits in the sensory cortex. Impaired LTD could lead to excessive synaptic strength and sensory hypersensitivity, while impaired LTP may prevent the formation or strengthening of connections necessary for normal sensory processing or integration (66). Many ASD-associated gene mutations are predicted to influence synaptic plasticity directly or indirectly, for example by dysregulating protein synthesis and degradation (2, 67).

A specific synaptic plasticity-related theory is the metabotropic glutamate receptor (mGluR) hypothesis for fragile X syndrome (68). In *Fmr1* null mice, mGluR-mediated LTD is exaggerated due to the absence of the fragile X protein (FXP, encoded by the *Fmr1* gene), leading to increased protein synthesis levels (69). Excessive LTD is associated with an increased prevalence of long, thin dendritic spines with weaker synapses, which may reflect immature circuits that generate weak or imprecise sensory codes. Other mouse models of ASD also have immature spine phenotypes and altered mGluR signaling and/or mGluR-LTD (70–72), and it appears that dysregulation of protein synthesis and mGluR-LTD in either direction may lead to ASD-related behaviors (73). Activity-dependent protein synthesis subserves both LTP and LTD and is dysregulated in numerous ASD models (74, 75). Decoupling protein synthesis from network activity will affect not only mGluR-mediated forms of plasticity but also have numerous consequences for sensory circuit development and function. Recent reviews discuss these synaptic plasticity and protein synthesis-related hypotheses in depth (76–78).

### Impaired critical period plasticity and synapse maturation

Sensory cortical areas undergo robust activity-dependent plasticity during critical periods in development, when environmental input drives synapse maturation and refines and stabilizes circuits. In the impaired critical period hypothesis, ASD pathophysiology disrupts critical period plasticity, perturbing normal circuit maturation to produce long-lasting changes in circuit organization and behavior (79). Such critical period disruption could be due to impairments in cellular plasticity mechanisms, abnormal sensory experience, or abnormal circuit activity patterns. Impaired or dysregulated synaptic plasticity rules are well described in several ASD mouse models (see “Abnormal synaptic plasticity”). In addition, PV hypofunction is likely to disrupt critical period plasticity because PV interneurons are critical for regulating the timing of critical periods. Because PV neurons tend to sharpen the sensory tuning of PYR cells, PV hypofunction could also impair the sensory-guided development of precise sensory circuits, undermining the development of typical sensory processing (80).

Children often begin showing behavioral signs of ASD during these critical windows of development, although structural and physiological changes in neural circuitry could begin as early as infancy (81). Multiple mouse models of ASD show disrupted critical periods (82–84), altered critical period timing (85), and critical period-related synaptic impairments that include impaired cellular plasticity, delayed maturation of inhibitory circuitry, and abnormal retention of immature dendritic spines and silent synapses (86–90). Whether these abnormalities reflect a specific deficit in critical period plasticity or a more general impairment of plasticity throughout life, remains unclear.

### Degraded sensory coding

The local circuit and synaptic alterations described above are all likely to lead to impairments in the neural coding of sensory information. Degraded coding could take many forms, including broadened sensory tuning of individual neurons, blurred sensory maps, reduced signal-to-noise ratio of sensory responses relative to spontaneous activity, increased trial-to-trial sensory response variability, and altered firing correlations that reduce information carried by population codes. We define degraded coding as changes in sensory coding that reduce the information available for sensory detection or discrimination. Such changes would dim, blur, or distort perception and could underlie both atypical sensory processing and downstream behavioral phenotypes such as sensory seeking, sensory aversion, or insistence on sameness. The degraded coding hypothesis supposes that the specific genetic, cellular, or circuit origin of the deficits is less relevant than the coding deficit itself, so that convergence across genetic forms of ASD occurs on the neural coding level.

Many mouse models of ASD exhibit degraded sensory coding in the form of reduced signal-to-noise for sensory responses (34, 53, 91, 92), increased trial-to-trial response variability (93–95), abnormal sensory maps (83, 96–98), degraded sensory tuning (40, 93–95, 99), or abnormal firing correlations (100–105). In people with ASD, studies have reported impaired sensory discrimination for touch and vision (40, 106, 107), impaired detection of speech in noise (21, 108), altered sensory-evoked event-related potentials (ERPs) (18), increased trial-to-trial variability of sensory-evoked ERPs (109), and altered topography of cortical sensory maps (110, 111). These suggest degraded sensory coding, though more quantitative psychophysical measurements are needed.

Degraded sensory coding is distinct from the classical E–I ratio theory, which proposes hyperexcitability and excess spiking in ASD, leading to sensory hypersensitivity (49) and an overly intense sensory world (52). Sensory hyper-reactivity and aversion in people with ASD may reflect either excessive psychophysical intensity or a heightened affective reaction but a normal perception of intensity (20, 47, 112). Across ASD mouse models, even those with increased E–I ratio in the sensory cortex, normal or below-normal neural sensory-evoked spiking is common (34, 40, 53, 91, 99, 113) while excess spiking is relatively rare (36, 94, 114). Thus, degraded coding may be more prevalent than hyperexcitability in the sensory cortex, at least in mice.

### Failure of sensory adaptation

Adaptation to repeated or continuous stimuli is a common feature of sensory processing and serves to increase coding efficiency and enhance the representation of novel stimuli by reducing spiking to repeated, predictable stimuli. This theory proposes that in ASD, cellular and circuit abnormalities cause a failure of adaptation so that repeated stimuli evoke abnormally strong cortical spiking. This may lead to sensory processing impairments, sensory hypersensitivity, and behavioral avoidance of ongoing stimuli. Failure of adaptation will preferentially affect coding in sensory contexts with dense ongoing stimuli, which are common in natural environments. Consistent with this theory, individuals with ASD can show reduced adaptation to audio-visual stimulation (115) and tactile stimuli (29, 107, 116).

In ASD mice, evidence for this theory comes from *Fmr1–/–* mice, which show impaired spike adaptation to repeated whisker stimuli in L2/3 of the S1 cortex and behavioral avoidance of repetitive whisker stimulation, termed ‘tactile defensiveness' (37). *Fmr1–/–* mice also have impaired habituation in A1 to repeated sounds (117). In *Fmr1–/–* mice, pharmacological or pharmacogenetic enhancement of PV cell spiking increases response adaptation by PYR cells to repeated whisker stimuli and reduces tactile defensiveness (61). This suggests that impaired adaptation in *Fmr1–/–* mice arises at least in part from PV hypofunction in the sensory cortex, although other mechanisms could also contribute. Whether other ASD models have impaired sensory adaptation is unknown, though *Ube3A m–/p*+ mice have changes in inhibitory synaptic transmission that potentially suggest changes in response adaptation (118).

### Oscillations and altered synchrony

Cortical sensory areas exhibit rhythmic oscillations at a wide range of frequencies (119). Both the amplitude and phase of oscillations, particularly in the gamma (>30 Hz) and alpha (8–12 Hz) bands, have been correlated with sensory perception in humans (120–125). In standard models, gamma rhythms (thought to be generated by PV interneuron networks) create a temporal scaffold for local sensory processing and information relay that is necessary for sensory perception, while alpha rhythms are part of an attentional suppression mechanism. EEG and magnetoencephalography (MEG) studies have reported atypical oscillations in both the gamma and alpha bands in humans with ASD (126). In multiple studies, visual-evoked gamma rhythm was weakened in individuals with ASD (127–134), suggesting a processing impairment. Yet other studies found no difference or increased gamma power (135–138), so there is no complete consensus (139). Some studies have shown weaker alpha power or aberrant alpha power modulation in ASD children, which may indicate impaired suppression of responses to irrelevant stimuli (129, 140). In addition to these local effects, synchronization between the left and right hemisphere V1 is reported to be disrupted across a wide range of frequencies (141–143), which may correlate with impaired perceptual integration (143).

Oscillations have been studied less in mouse models of ASD. Divergent effects on gamma power in the auditory cortex have been reported, from reduced (138, 144, 145), to increased (146), to not different from wild type (147). Some studies have reported that gamma phase locking is impaired (138, 144, 145). Thus, gamma oscillations may be abnormal in sensory cortex in humans with ASD, and interhemispheric coherence and gamma phase locking may be reduced, but whether these effects are reliably observed in ASD mouse models remains unclear. More work needs to be done to discern common oscillation and synchrony phenotypes in mice and to identify the underlying circuit mechanisms.

## Convergence across mouse models of autism

Here, we examine the evidence for each of these circuit-level theories across ASD mouse models to evaluate the extent to which distinct genetic forms of ASD converge on any of these circuit-level deficits. Of the theories summarized above, sufficient empirical studies exist across mouse models to begin to evaluate convergence for four theories. These are the E–I ratio and hyperexcitability hypothesis, the PV hypofunction hypothesis, the impaired homeostasis hypothesis, and the degraded sensory coding hypothesis.

### E–I ratio and hyperexcitability

Elevated E–I ratio is theorized to drive hyperexcitability and excess spiking in the sensory cortex in ASD mouse models, leading to behavioral hypersensitivity. At the local circuit level, synaptic E–I ratio is demonstrably increased across many ASD models, including in S1 for *Fmr1–/–, Cntnap2–/–, Tsc2*+*/–*, and *16p11.2* deletion (53), driven by a large reduction in synaptic inhibition coupled to a weaker drop in excitation onto L2/3 pyramidal cells. Remarkably similar synaptic findings are reported in V1 for *Mecp2* null and *Ube3A m–/p*+ mouse lines (93, 118). However, rather than cause excess spiking, the increase in E–I ratio in the four mouse models from Antoine et al. (53) was shown to quantitatively predict normal net synaptic depolarization and to be associated with normal firing rate in L2/3 pyramidal cells measured *in vivo*. This shows that while an increased E–I ratio is common, it does not necessarily drive hyperactive cortical circuits in ASD mice.

There is convincing evidence for neural hyperactivity in primary sensory cortices in just three mouse lines: *Scn1a*+*/–, Shank3B–/–*, and some cortical areas in *Fmr1–/–*. *Scn1a* is a major sodium channel in forebrain GABAergic interneurons, and *Scn1a*+*/–* mice show reduced excitability of PV and somatostatin interneurons that reduces recurrent inhibition and leads to strong hyperexcitability and epilepsy, including in S1 (148–152). *Shank3B–/–* mice have increased spontaneous and whisker-evoked calcium responses in L2/3 pyramidal neurons in S1 and reduced activity in interneurons, and this excess pyramidal activity is associated with behavioral hyper-reactivity to weak whisker stimulation in a whisker detection task (36). Spontaneous spiking in L5 of S1 is also strongly elevated in *Shank3B–/–* (153) and hyperexcitability is present in V1 (154). However, one study did not observe neural hyperactivity in S1 using *c-fos* (33). *Fmr1–/–* mice show excess sensory-evoked spiking in some sensory areas, including in A1 and forepaw S1 (94, 114). These mice show audiogenic seizures, but these are due to circuit hyperexcitability in the inferior colliculus, not the auditory cortex (155). In slice physiology experiments in *Fmr1–/–* mice, thalamocortical circuits evoke sustained up-states in S1, indicating local circuit hyperexcitability (156). Together, these results indicate hyperactivity in A1 and S1 in *Fmr1–/–* mice. However, hyperexcitability is not present in *Fmr1–/y* in other sensory areas, for example in whisker S1, where spiking to preferred whisker stimuli is normal or slightly depressed with broadened single-neuron tuning, leading to blurred somatotopic maps (53, 97, 157). Excess spikes are also not apparent in V1 (40). Thus, *Fmr1–/y* mice appear to show hyperexcitability only in some cortical areas.

*Cntnap2–/–* mouse models present somewhat weaker evidence for hyperexcitability, which is inconsistent across studies. *Cntnap2–/–* mice show increased *c-fos* expression in S1 following whisker stimulation (158), but no change in spontaneous or whisker-evoked spiking in S1 measured with extracellular recordings (53). In V1, neurons are hyporesponsive to visual stimuli, and mice show behavioral hyposensitivity and impaired discrimination (41). In A1, *Cntnap2–/–* mice show reduced spontaneous activity and slightly increased sound-evoked spiking responses (159), which are associated behaviorally with increased startle and impaired auditory filtering (160). Thus, *Cntnap2–/–* rodents show a range of spike rate phenotypes but not consistent evidence for excess spiking.

Other mouse lines, including *Mecp2* and *Syngap1*, show substantial evidence of decreased cortical excitability. In both *Mecp2* null and *Mecp2* duplication mice, multiple reports show a decreased E–I ratio, involving both reduced synaptic excitation (91) and increased inhibition associated with increased PV cell number and/or PV expression in V1, S1, and A1 (85, 161, 162). These changes are associated with a strong reduction in sensory-evoked neural responses in V1 (43, 45, 163). A different study in *Mecp2* null mice found an increased E–I ratio in V1 as a result of a preferential reduction of inhibition over excitation, but still observed abnormally weak visual-evoked spiking (93). In contrast, A1 of *Mecp2* knockout mice shows hyperexcitable auditory responses (164). The reason for discrepancies between various *Mecp2* studies is not clear but could be related to age differences (163, 165). Hypoactivity is also evident in *Syngap1*+*/–* mice, which displayed reduced whisker-evoked activity in L2/3 pyramidal cells in S1, due to reduced whisker-evoked synaptic input and reduced intrinsic excitability (34). This neural hypoactivity was correlated with poor performance in tactile object detection and discrimination.

**Figure 2:**
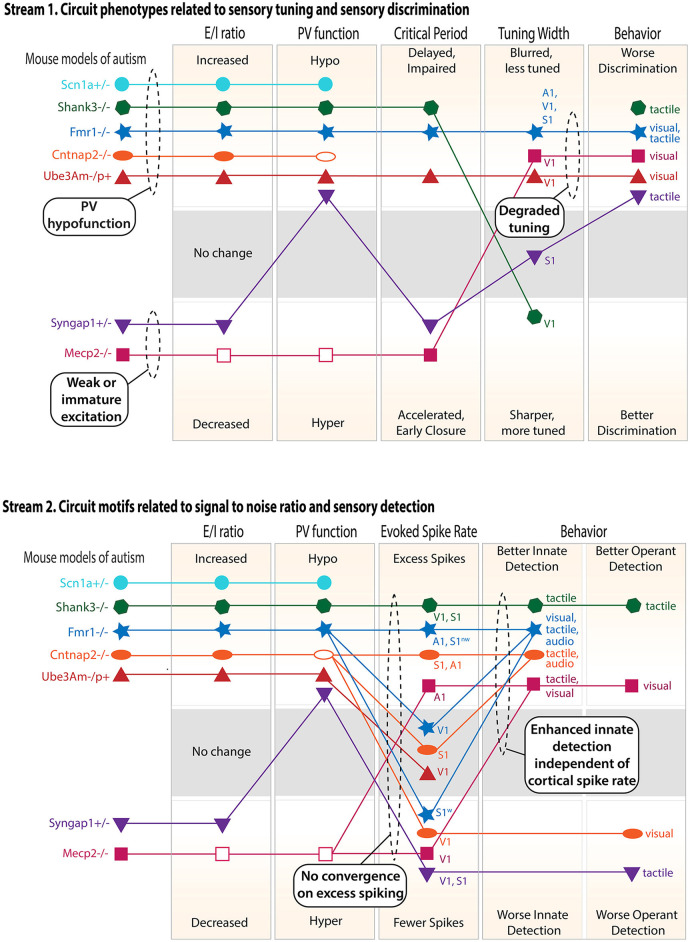
Cluster analysis of the circuit, neural coding, and behavioral phenotypes across different genetic mouse models of ASD. Only findings from V1, S1, and A1 are considered, and only mouse models with a substantial number of published studies are included. **Top panel**: circuit phenotypes related to sensory tuning and sensory discrimination behavior. Discrimination behavior results include both innate discrimination behavior and operantly trained discrimination behavior (see text). Within each rectangle, the axis is organized so that the naively predicted consequences of a high E–I ratio are above and the consequences of a low E–I ratio are below. When multiple studies in the same cortical area gave conflicting results, an unfilled symbol was used to indicate the major reported effect. When multiple studies in different sensory regions or modalities gave different results, multiple points were plotted and labeled accordingly. Lines show relationships within each mouse model. S1^w^ denotes whisker S1, and S1^nw^ denotes non-whisker S1. **Bottom panel**: circuit phenotypes related to signal-to-noise ratio and sensory detection behaviors. Dashed ovals highlight convergence across ASD models or logical conclusions that can be drawn across models.

Thus, *Shank3B–/–, Scn1a*+*/–*, and *Fmr1–/–* in some brain regions form a phenotypic cluster that exhibits increased E–I ratio and excess spiking and often correlates with hypersensitivity in sensory detection tasks (Figure 2). In other sensory areas, *Fmr1–/–* has normal or reduced spiking, rather than increased spiking. *Cntnap2–/–* shows mixed results, and in at least one study, *Cntnap2–/–* is similar to *Fmr1–/y, 16p11.2* del, and *Tsc2*+*/–* mice in showing elevated synaptic E–I ratio but no increased spiking (53), with *Ube3a m–/p*+ showing similar results. These latter genotypes may have abnormal cortical circuit function due to reduced inhibition, but they do not exhibit overt hyperexcitability. In contrast, *Syngap1*+*/–* and most *Mecp2* null studies indicate a second phenotypic cluster, which generally shows a reduced E–I ratio and reduced spiking in the sensory cortex. Both clusters exhibit impairments in sensory processing (described below), but only the first cluster shows excess spiking.

Therefore, the expectation that an increased E–I ratio drives excess spiking and that this leads to sensory hypersensitivity holds only rarely, and just for genes in the first cluster (Figure 2). These represent ASD genes that are essential for interneuron function but have a smaller role in PYR cells (e.g., *Scn1a*, which encodes Na_V_1.1, the main voltage-gated sodium channel in cortical interneurons). Loss of function of these genes is likely to weaken inhibition and increase the E–I ratio very substantially without a sufficient compensating drop in excitation, thus driving excess PYR spiking. Other ASD genes are associated with a modestly elevated E–I ratio, which appears to degrade neural coding without an elevation of net spiking in the sensory cortex, or a decreased E–I ratio, which correlates with reduced spiking (as in the case of the *Syngap1*+*/–* and *Mecp2* null clusters) (Figure 2). Overall, increased E–I ratio does not systematically predict elevated spike rates. This may be because in many cases, a modest increase in E–I ratio associated with normal spike rates may actually reflect an endogenous homeostatic adjustment of E–I ratio that is recruited to preserve the mean firing rate or other aspects of sensory processing (53). Consistent with these results from mice, an analysis of syndromic ASD individuals shows that cortical hyperactivity vs. hypoactivity does not correlate well with sensory hypersensitivity vs. hyposensitivity, arguing further against the monolithic E–I ratio hypothesis (166).

### Hypofunctional parvalbumin inhibitory circuits

Parvalbumin (PV) circuit dysfunction is strongly linked to autism, both in people with ASD and across genetically distinct ASD mouse models (54). PV is a calcium-binding protein whose expression in PV interneurons is believed to be activity-dependent (167). PV cell number, PV expression level, and PV circuit function have all been shown to be perturbed in ASD (58). Given the many impacts of PV interneurons on circuit function and neural coding, including sharpening sensory tuning, regulating E–I ratio and sensory response gain, contributing to sensory adaptation, and generating gamma oscillations, PV circuit dysfunction could drive multiple impairments in sensory processing and perception (80).

Reduced PV cell number, assayed by anti-PV immunostaining, is observed in sensory cortex in many ASD models, including *Fmr1–/–* (61, 168, 169), *Shank3B–/–* (170), *Neuroligin-3–/–* (171), *Arid1b*+*/–* (172), and some studies of *Cntnap2–/–* (173, 174) but not others (175, 176). In S1, PV loss in *Shank3B–/–* and *Cntnap2–/–* mice is strongest in the hemisphere corresponding to the mouse's dominant paw (177). Loss of PV cells could be due to their failure to develop properly or through selective apoptosis. Both of these factors are known to contribute to GABAergic cell loss in *Arid1b*+*/–* mice (172). In S1 of *Fmr1–/–* mice, PV cells die off via apoptosis during early postnatal development due to insufficient PV cell activity (61). In other ASD models, it remains unclear whether PV cell number is truly reduced or whether PV cell hypoactivity causes PV protein expression to fall below detectable levels (170). The use of alternative markers for PV cells, such as labels targeting the perineuronal net, can resolve this issue.

Those PV cells that remain are hypoactive in many mouse models. In S1 slices, feedforward L4-L2/3 inhibition, which is known to be PV-mediated, is reduced in *Fmr1–/–, Cntnap2–/–, 16p11.2 del*, and *Tsc2*+*/–* mice, and whisker stimuli evoke 50% fewer spikes than normal in L2/3 fast-spiking (presumed PV) units in *Fmr1–/–, Cntnap2–/–*, and *16p11.2 del* mice (53). Whisker-evoked spiking in PV cells is also greatly reduced in *Syngap1*+*/–* mice (34) and *Shank3B–/–* mice (36). In V1, reduced visual-evoked activity is observed in PV interneurons in *Fmr1–/–* mice (40) and in one study in *Mecp2* null mice (93). Reduced sensory responses in PV cells can have diverse causes, including reduced PV intrinsic excitability in *Tsc2*+*/–* mice (178) and *Scn1a*+*/–* mice (148), and delayed development of intrinsic excitability and excitatory synaptic input to PV cells in *Fmr1–/–* mice (89, 156).

The prediction in the cases above is that PV hypofunction would promote excess spiking, but as discussed in the “E–I ratio and hyperexcitability” section, this is not always true. Instead, PV hypofunction can lead to either network hyperexcitability or other types of downstream effects, including dysregulation of critical period plasticity, broadening of sensory tuning in PYR cells, or failure of sensory adaptation, as discussed below. Two clear examples of this divergence are *Shank3B–/–* and *Fmr1–/–* mice, which both exhibit PV hypofunction and increased E–I ratio. In *Shank3B-/–*, this leads to excess spiking in S1 and enhanced tactile detection (36, 40), but in *Fmr1–/–*, this leads to variable findings of excess, normal, or slightly reduced spiking in S1 (53, 97, 114, 157), and no excess spiking in V1 but broadened orientation tuning of PYR cells that impairs behavioral discrimination (36, 40).

Unlike the mice above that show PV hypofunction, *Mecp2* null mice generally exhibit PV hyperfunction, which contributes to an overall increase in inhibition in the sensory cortex, particularly in the juvenile period. These mice show elevated PV expression and excitatory synaptic input to PV cells in S1 and excess PV synapses and PV inhibitory transmission in V1 (85, 162, 163). Some evidence suggests early PV hyperfunction may give way to PV hypofunction in adulthood (93, 163).

Because PV cells regulate critical periods in the sensory cortex, a reduction in PV number or a delay in PV circuit maturation could drive consequential changes in the developmental refinement of neural circuitry. This has been examined most closely in *Fmr1* null mice, where PV interneurons in S1 show delayed development of intrinsic excitability and synaptic connections that are rescued by treatment with a TrkB agonist (89, 168). Delayed PV maturation also occurs in A1, where it involves delayed perineuronal net formation, the rescue of which is sufficient to improve network hyperactivity (169). In S1, *Fmr1* null mice exhibit a delayed developmental transition from depolarizing to hyperpolarizing GABA, and correcting this imbalance restores the precision of the somatosensory map (179). Critical period plasticity is also impaired in *Ube3A m–/p*+ mice (84). In contrast, *Mecp2*+*/–* mice show an accelerated critical period in the visual cortex, which is attributed to elevated GABAergic activity (85, 161).

Given broad GABAergic changes in ASD brains, multiple studies have focused on manipulating all or broad subsets of interneuron types (MGE-derived vs. CGE-derived) to determine their role in ASD phenotypes. Deletion of *Shank3B* from forebrain interneurons is sufficient to drive whisker hypersensitivity, as is acutely reducing inhibition using chemogenetics in wild-type mice (36). *Scn1a* is primarily expressed in interneurons, and its selective deletion from *Dlx1/2*+ interneurons recapitulates the full knockout ASD phenotype (148). In maternal *Ube3A m–/p*+ mice, re-expression of *Ube3A* selectively in GABAergic cells corrects increased spiking and improves orientation tuning deficits (99). It is unknown whether these effects are due to effects on PV cells alone or other interneuron types.

Specific manipulation of the PV interneuron population has been extensively studied in *Fmr1–/–* mice across sensory areas. Activation of PV cells using chemogenetics restored visual responsiveness and orientation tuning and improved performance on an orientation discrimination task (40). Similarly, in S1 of *Fmr1–/–* mice, pharmacological or chemogenetic activation of PV cells restored normal sensory adaptation in pyramidal cells and rescued tactile defensiveness behavior (61). Interestingly, *Fmr1* re-expression selectively in excitatory neurons during P14–P21 was sufficient to rescue the reduction in PV cell density and activation in A1 (180), suggesting *Fmr1* mutation in excitatory neurons drives changes in inhibitory circuits. Haploinsufficiency of the PV gene itself causes autism-like behaviors and neural activity phenotypes, and rescuing PV levels is sufficient to rescue social behavior (181, 182). Thus, selective loss or restoration of ASD genes in PV cells or GABAergic interneurons can directly drive or rescue ASD circuits and behavioral phenotypes.

Whether *Mecp2* acts exclusively in PV neurons to drive ASD-related phenotypes is unclear because of conflicting results in the literature. In some studies, *Mecp2* loss from GABAergic neurons recapitulates Rett syndrome features and reduces levels of GABA synthesis enzymes (93, 183). PV-specific deletion of *Mecp2* resulted in a decreased evoked spike rate in PV cells upon visual stimulation, indicating PV hypofunction and impaired V1 critical period plasticity (88). These studies are consistent with the idea that PV hypofunction (either caused cell-autonomously by *Mecp2* loss in PV cells or secondary to loss in excitatory cells) can contribute to Rett phenotypes. But in another study, deletion of *Mecp2* from forebrain excitatory but not inhibitory neurons led to seizures and a cell-autonomous reduction in GABAergic transmission, suggesting *Mecp2* loss has its primary effect in excitatory neurons (184). Mecp2 is also expressed in other interneuron types, where its deletion may have complementary effects (185).

In summary, PV circuit hypofunction is a convincing feature of many ASD models, including *Fmr1–/–, Shank3B–/–, Cntnap2–/–, Scn1a*+*/–*, and *Ube3A m–/p*+ (Figure 2). This can be due to the loss of PV cells, decreased PV synaptic connectivity, reduced PV intrinsic excitability, or reduced PV expression itself. In some cases, PV hypofunction is driven by mutations in genes acting in PV cells specifically, while in other cases it may be a secondary, adaptive response to alterations in excitatory networks, e.g., via PV circuit homeostasis. PV hypofunction does not manifest in the same circuit- or sensory deficits in all ASD mouse models, and instead drives a variable set of circuit consequences ranging from excess spiking in PYR cells (*Shank3B, Scn1a*, some *Fmr1* studies) to broader sensory tuning in PYR cells without excess spiking (other *Fmr1* studies) to delayed or impaired critical periods (*Fmr1–/–, Ube3A m–/p*+). *Mecp2* studies are mixed, but some suggest that *Mecp2* null mice exhibit enhanced, not reduced, PV circuit function.

In spite of strong evidence for GABAergic dysfunction in humans with ASD (186) and successful rescue of ASD-related behaviors in mice using GABA modulators or PV circuit modulation (detailed above), clinical trials using GABAergic modulators to correct inhibition have not shown a substantial effect in treating sensory issues in children with ASD (59, 187). A possible explanation is that circuit dysfunction specifically reflects the hypofunction of PV cells and not other interneurons, suggesting that therapies should focus on PV-specific modulation. However, the relative contribution of early developmental PV impairment (which impairs critical period circuit refinement) vs. ongoing adult PV circuit impairment for ASD phenotypes remains unknown. Thus, new approaches using chemogenetics, photoactivatable proteins, small molecules, and gene therapies to selectively target PV cells in precise time windows may prove useful in treating ASDs and other neuropsychiatric disorders (188, 189).

### Altered homeostasis

Impaired homeostatic plasticity has been theorized to underlie ASD, but studies evaluating this hypothesis are still somewhat scarce in the sensory cortex. Many ASD genes are involved in the activity-dependent regulation of network excitability (64), so the breakdown of homeostatic plasticity in ASD is a plausible mechanism of sensory circuit dysfunction.

Synaptic scaling is the most well-characterized homeostatic plasticity mechanism and has been extensively studied in ASD. Synaptic scaling is impaired in many mouse models, including *Fmr1–/–, Mecp2–/–, Cntnap2–/–, Syngap1*+*/–*, and *Shank3B–/–* (190–194). The classical form of synaptic scaling is up-scaling of excitatory synapses, induced in cultured neurons in response to silencing the network with TTX or glutamatergic blockers. In up-scaling, the synaptic strength of excitatory synapses is increased due to the insertion of α-amino-3-hydroxy-5-methyl-4-isoxazolepropionic acid receptor (AMPAR). Up-scaling is absent in neuronal cultures from V1 of *Shank3B–/–* mice (194), and is reduced in cultures from *Cntnap2–/–* and *Mecp2* knockdown neurons (190, 191). Synaptic scaling may fail for *Shank3B–/–* and *Cntnap2–/–* due to the role of these genes as postsynaptic scaffolding molecules and for *Mecp2* because of its role as a transcriptional regulator. Interestingly, *Fmr1–/–* mice have impaired excitatory and GABAergic synaptic scaling in cultures from the hippocampus (192, 193), but synaptic scaling is normal in cultures from the cortex (195), which is surprising given that the fragile X protein is a key regulator of AMPAR receptor transcription and translation (196).

Homeostatic plasticity of intrinsic excitability is also induced by network silencing in neuronal cultures and is impaired in two ASD mouse models, *Shank3B–/–* (194) and *Fmr1–/–* (195). This may reflect direct and indirect interactions between Shank3 and FXP with ion channels that are regulated during homeostatic intrinsic plasticity. Whether other ASD mouse models show similar deficits has not been tested.

*In vivo*, homeostatic synaptic scaling, homeostatic plasticity of intrinsic excitability in pyramidal cells, and homeostatic plasticity in PV circuits work together to actively stabilize the mean firing rate of cortical PYR cells, which can be observed experimentally in response to sensory manipulations. For example, sustained monocular visual deprivation causes an initial rapid reduction in firing rate in PYR cells in V1, due to Hebbian plasticity mechanisms that suppress cortical responses to the closed eye, but after several days, firing rates begin to climb to restore pre-deprivation firing rates (197). This restoration, termed “firing rate homeostasis,” is due to homeostatic synaptic scaling, homeostatic intrinsic plasticity, and downregulation of PV circuit activity (198–200). Similar mechanisms occur in S1 to maintain stable firing rates for several days after whisker deprivation (201–203). In *Shank3B–/–* mice, firing rate homeostasis is abolished or substantially delayed (194). Visual deprivation also fails to induce synaptic scaling in V1 *in vivo* in *Mecp2–/–* and *Cntnap2–/–* mice (190, 191). Whether PV circuit homeostasis is impaired in ASD remains unknown, but it could be an important aspect of cortical pathophysiology that destabilizes pyramidal firing rates and sensory coding. Thus, more studies are needed, but existing evidence points to a major impairment in homeostatic plasticity in V1 across several ASD models.

### Degraded sensory coding

Population imaging of neural activity and high-density single-unit recording in ASD mouse models provide strong evidence for neural coding disruptions in the sensory cortex. As discussed above, relatively few ASD mouse models show excess sensory-evoked spiking. Instead, mean sensory-evoked spike rate is often similar to wild type, as in whisker S1 of *Cntnap2–/–, 16p11.2 del*, and *Tsc2*+*/–* mice (53), V1 of *Fmr1–/–* mice and *Ube3a m–/p*+ mice (40, 118), or is weaker than wild type, as in S1 of *Syngap1*+*/–* mice and *Fmr1–/–* mice (34, 53), and V1 of *Mecp2* null mice (93). But multiple aspects of neural coding are abnormal in these cases, including elevated spontaneous firing, increased trial-to-trial variability, broadened sensory tuning, blurred sensory maps, and abnormal adaptation. These coding phenotypes likely reflect cortical circuit dysfunction and may contribute to altered sensory behavior in ASD.

Spontaneous firing represents baseline noise in the absence of sensory stimuli and affects the signal-to-noise ratio for stimulus encoding. Some ASD models exhibit altered spontaneous activity, with *Shank3B–/–* mice exhibiting increased and *Syngap1*+*/–* mice exhibiting decreased spontaneous activity in S1 (34, 36). In contrast, spontaneous firing is largely normal in S1 and V1 in *Fmr1* and *En2* null mice (37, 40, 53, 83, 97, 114). Trial-to-trial reliability of sensory-evoked responses also affects coding accuracy and thus impacts behavioral detection and discrimination of stimuli. Trial-to-trial variability in sensory responses is increased in A1 and V1 of *Fmr1, Cacna2d3*, and *Mecp2* null mice (93–95), which is predicted to worsen sensory performance. This is consistent with excess trial-to-trial variability measured in visual, auditory, and somatosensory-evoked ERPs in people with ASD (109).

Degraded sensory tuning of single neurons (i.e., broader tuning) will also degrade sensory perception, by blurring differences in population activity across different stimuli. In *Fmr1–/–* mice, frequency tuning in A1 (94), whisker tuning in L2/3 of S1 (97), and orientation tuning in L2/3 of V1 (40) are all broader compared to the control. For *Mecp2*, two studies in *Mecp2–/–* and *Mecp2* overexpression mice found broader tuning in V1 (43, 93), but other studies found normal tuning width (45, 85). Broader orientation tuning was also found in V1 of *Ube3a m–/p*+ mice (99), and broader whisker tuning in S1 of *15q duplication* mice (92). Thus, broadened sensory tuning is a common motif across S1, A1, and V1 (Figure 2). But this phenotype is not universal, with *PTEN* and *Cacna2d3–/–* mice showing normal sensory tuning (95, 204), and *Shank3*+*/–* mice showing overly narrow orientation tuning in V1 (205). Broader tuning is expected to correlate with impaired behavioral discrimination of sensory stimuli, and such impairments have been observed for tactile stimuli (33, 34, 206), and for visual orientation (40) across several ASD mouse models.

Topographic maps are another important feature of primary sensory cortices and are also impacted in multiple ASD mouse models. The somatotopic whisker map is blurred in *Fmr1–/–* mice (53, 97, 157, 179), and the tonotopic map in the auditory cortex is blurred in *Mecp2* overexpression mice (98), *Fmr1–/–* mice (94) and in the VPA rat model (96). The binocular area of V1 is expanded in *En2* null mice (83). These examples of blurring or altered map topography are consistent with alterations in map topography in humans with ASD (110, 111). Thus, ASD mouse models commonly show broadened single-neuron sensory tuning and blurred or altered sensory maps. These phenotypes are likely to result in impairments in sensory discrimination.

Other aspects of neural coding are also disrupted in multiple ASD mouse models, including abnormal sensory adaptation in *Fmr1–/–* mice (37), which can be rescued by PV cell activation (61), and abnormal firing synchrony between neurons in local networks in *Fmr1* and *Cntnap2* null mice (100–105). Decoding of sensory information by downstream regions may be impaired by abnormal activity correlations across areas, which have been observed in *Shank3* null macaques and in humans with ASD (207, 208), and abnormal gamma rhythms, as observed in *Fmr1* and *Cntnap2* null mice and in people with fragile X syndrome (104, 144, 145, 180, 209).

Thus, degraded neural coding is evident on multiple levels across many mouse models, even when sensory-evoked firing rates are near normal. These coding deficits are predicted to reduce the amount of sensory information available for detection and discrimination at the level of the primary sensory cortex, which may create a dim, blurred, or confusing sensory world. Across ASD mice, there is no single uniform type of coding degradation, but degraded sensory tuning and blurred maps are the most common. These phenotypes are predicted either from an acute lack of PV inhibition that normally sharpens sensory tuning or from a developmental failure of experience-dependent strengthening, refinement, and consolidation of synapses during critical periods. In the latter case, sensory enrichment or training that engages natural plasticity mechanisms could potentially restore coding precision, perhaps in concert with treatments that enhance the capacity for synaptic plasticity.

### What are we missing beyond the primary sensory cortex?

In this review, we have only considered circuit and coding abnormalities in the primary sensory cortex, but other brain areas are also likely to contribute to ASD sensory phenotypes. For example, multiple ASD mouse models show hyperexcitability in peripheral somatosensory receptor neurons that contribute to behavioral touch hypersensitivity and may drive downstream circuit changes in the somatosensory cortex, including homeostatic changes to compensate for the increased upstream sensory drive (35). These peripheral sensory changes could also drive secondary social behavioral impairments due to distorted or aversive touch-related social cues during critical periods for social behavior (27). Other subcortical sensory circuits could contribute to elevated sensory responsiveness in ASD, potentially including the brainstem, amygdala, or cerebellum (which builds ongoing predictive models of sensory input).

We also did not review theories of abnormal functional connectivity between brain areas, which have been suggested by fMRI and EEG studies in people with ASD. Such abnormalities could alter the propagation and integration of sensory information in the brain, but they include a wide variety of phenotypes across brain areas and ages (210) and are currently difficult to map onto underlying circuit mechanisms. One interesting form of this theory is that sensory impairments in autism arise from atypical hierarchical processing, in which there is an over-reliance on bottom-up sensory processing and weakened top-down modulation, leading to impaired ability to contextualize sensory input (31, 211). While this idea has some interesting support (212), the prevalence, circuit mechanisms, and functional consequences remain unclear.

## Synthesis: convergence and clustering across ASD mouse models

The circuit, coding, and behavioral phenotypes for the best-studied ASD mouse models presented above are plotted in Figure 2 as a means of visualizing convergence and clustering across mouse models. This analysis also allows predictive relationships between theories to be visualized. Results are separated into two logical streams in order to better visualize associations. Each stream focuses on a different potential effect of inhibition on circuit function, neural coding, and sensory behavior.

### Stream 1—circuit properties related to sensory tuning and discrimination

PV circuits impact the sensory tuning of pyramidal cells. This occurs both during development, where PV inhibition gates the critical periods that refine excitatory circuits to create appropriate sensory tuning and sensory maps, and in the adult brain, where PV inhibition is recruited by sensory stimuli and acts to acutely sharpen PYR cell sensory tuning. Thus, abnormal PV circuit function in ASD, either in development or adulthood, may be expected to alter the sensory tuning of pyramidal cells, which in turn is likely to impact sensory discrimination behavior. To test for these relationships, the first stream plots the E–I ratio, PV function, critical periods, sensory tuning, and sensory discrimination behavior, including both innate (e.g., texture novel object recognition) and operantly trained discrimination behavior.

Key lessons from this analysis:

1. “PV hypofunction” and “weak or immature excitation” clusters. By examining E–I ratio, PV circuit function, and critical period plasticity, two clusters of mouse models emerge. the first cluster (*Shank3B–/–, Fmr1–/–, Ube3Am–/p*+, and *Cntnap2–/–*) shows elevated E–I ratio, weakened PV circuits, and delayed or impaired critical periods, which is a predicted consequence of PV hypofunction. This may be considered a “PV hypofunction” cluster, and *Scn1a*+*/–* is likely to also be in this cluster. The second cluster (*Mecp2–/–* and *Syngap1*+*/–*) shows the opposite effects: decreased E–I ratio and accelerated early closure of critical periods, sometimes with hyperfunction of PV cells (*Mecp2–/–*), but not always (*Syngap1*+*/–*). This may be considered a “weak or immature excitation” cluster.
2. All models show impaired sensory discrimination behavior. This behavioral phenotype can arise through different circuit mechanisms because it is associated with both PV hypofunction and weak excitation clusters.
3. Degraded sensory tuning is common and predicts impaired discrimination behavior. Multiple ASD models show degraded sensory tuning in the sensory cortex (and also blurred maps, not explicitly plotted in the figure). All of these models show impaired sensory discrimination. These are likely to be causally linked because degraded tuning reduces the information available in neural population codes to support sensory discrimination, and circuit manipulation in the primary sensory cortex that restores neural tuning can rescue behavioral discrimination phenotypes (40). Mouse models from both the PV hypofunction cluster and the weak excitation cluster can show degraded sensory coding, suggesting this is a common failure mode of cortical development or computation.

### Stream 2—circuit properties related to signal-to-noise ratio and sensory detection

PV circuits, and inhibition more generally, also impact spontaneous firing and the gain and signal-to-noise ratio of sensory-evoked responses. These aspects of neural coding are essential for stimulus detection. Thus, abnormal PV circuit function or abnormal inhibition in ASD may be expected to alter sensory detection behavior (for example, to generate sensory hypersensitivity, as in the original E–I ratio hypothesis). To test for these relationships, the second stream plots the E–I ratio, PV function, sensory-evoked spike rate, and sensory detection behavior, including both innate sensory detection behavior (e.g., paw withdrawal to a calibrated touch stimulus or acoustic startle) and operantly trained detection behavior.

Key lessons from this analysis:

1. Elevated E–I ratio and PV hypofunction do not predict excess PYR spiking. Instead, some models show excess spiking (although not in all studies or all brain areas), and others show normal or decreased spiking. The strongest or most consistent excess spiking phenotypes are in *Shank3*+*/–* and in some but not all sensory cortical areas in *Fmr1–/y*. *Scn1a*+*/–* has highly elevated spontaneous activity and is also likely to exhibit excess spiking, but this has not been tested. Other models show clear evidence for normal or decreased spiking. Among the mouse models with reduced E–I ratio (i.e., those in the “weak or immature excitation” cluster (*Syngap1*+*/–* and *Mecp2–/–*), there is a trend toward lower PYR spiking, but this is also not absolute. Thus, while some phenotypic clustering exists at the level of E-I ratio and PV function, there is no clear convergence at the level of PYR firing rates in the sensory cortex. Those models that show excess spiking may be those in which gene mutation decreases PV and other interneuron functions most dramatically, perhaps via direct regulation of interneuron intrinsic excitability (e.g., *Shank3B*+*/–* and *Scn1a*+*/–*), or in which gene mutation also impairs synaptic and cellular homeostasis, so that firing rate cannot be normalized by homeostatic mechanisms (e.g., *Fmr1–/y* and *Shank3B–/–*).
2. Innate sensory detection behavior is uniformly elevated, whether the sensory-evoked firing rate is increased or not. Innate detection behavior is enhanced across all ASD models in this review (i.e., lower detection thresholds or heightened behavioral responses). This occurs independent of whether the sensory-evoked firing rate is elevated in the sensory cortex or not. We suggest that this is because innate detection behaviors are often not cortically dependent, so this enhancement may be driven by subcortical circuit alterations in ASD (35), or by the breakdown of cortical modulation of subcortical circuits. Operant sensory detection behavior is more variable, sometimes being enhanced (e.g., *Shank3B–/–*) and sometimes being reduced (e.g., *Syngap1*+*/–* and *Cntnap2–/–*). While operant sensory detection behavior correlates well with PYR spike rates in the sensory cortex, innate sensory detection behavior does not. Overall, only a few mouse models show consistent elevation (*Shank3B–/–*) or reduction (*Syngap1*+*/–*) at all three levels of E–I ratio, PYR spiking, and sensory behavioral detection.

Strikingly, the literature reveals a consistent pattern of degraded sensory discrimination behavior but enhanced innate detection behavior across all ASD mouse models that have been tested. Why might this pattern exist? We hypothesize that because sensory discrimination behavior is typically cortically mediated, impaired discrimination reflects degraded sensory coding that is common in the sensory cortex across many ASD models. In contrast, innate detection behavior is often not cortically dependent, and Stream 2 shows that innate detection phenotypes do not correlate with spiking phenotypes in the sensory cortex. Thus, enhanced sensitivity for innate detection behaviors may not reflect excess cortical spiking but hypersensitivity of subcortical sensory pathways, which is known to occur in several ASD models (35). These severe syndromic forms of ASD may therefore reflect degraded cortical function, with the enhancement of subcortically driven behaviors due to loss of cortical modulation. Alternatively, discrimination behavior could be impaired simply because discrimination is more processing-intensive than detection and would be more affected by any minimal circuit dysfunction.

This meta-analysis reveals common circuit and sensory features across ASD mouse models and the presence of phenotypic clusters based on neurophysiology that can be labeled as “PV hypofunction” vs. “weak/immature excitation.” Ideally, future research will attempt to fill in the gaps across these mouse models and identify other patterns of convergence. Notably, recent studies are attempting to address this problem in individuals with autism by integrating large genetic datasets, functional connectivity studies, and sensory behavioral profiles to determine whether the expression of ASD-related genes can predict clusters of neuropathophysiology and behavior (15, 213, 214). Such approaches leverage the massive amounts of data available and newly developed machine learning tools but rely heavily on being able to compare and normalize data across studies (215). Thus, a key aspect of future research in human and mouse models is the creation and usage of standardized quantitative sensory behavior assays that integrate and compare naturalistic stimuli with social and non-social valence. Understanding convergence and divergence is decidedly useful from a therapeutic standpoint, as it may allow us to predict which individuals will benefit from which therapies. Moreover, by identifying shared domains of impairment in syndromic models the autism field can establish biomarkers of different forms of circuit impairment, which could be applied to stratify candidates with idiopathic autism into particular clusters of ASD pathophysiology and inform treatments options.

## Author contributions

HM: Writing—original draft. HW: Writing—original draft. DF: Funding acquisition, Writing—review and editing.

## References

[1] Ramaswami G Geschwind DH. “Chapter 21 - Genetics of autism spectrum disorder” In:Geschwind DH Paulson HL Klein C, editors. Handbook of Clinical Neurology. Neurogenetics, Part I. Elsevier (2018). p. 321–329. 10.1016/B978-0-444-63233-3.00021-X 29325621

[2] Huguet G Ey E Bourgeron T. The genetic landscapes of autism spectrum disorders. Annu Rev Genomics Hum Genet. (2013) 14:191–213. 10.1146/annurev-genom-091212-153431 23875794

[3] Levy SE Mandell DS Schultz RT. Autism. Lancet. (2009) 374:1627–38. 10.1016/S0140-6736(09)61376-3 19819542 PMC2863325

[4] Marco EJ Hinkley LBN Hill SS Nagarajan SS. Sensory processing in autism: a review of neurophysiologic findings. Pediatr Res. (2011) 69:48–54. 10.1203/PDR.0b013e3182130c54 21289533 PMC3086654

[5] Robertson CE Baron-Cohen S. Sensory perception in autism. Nat Rev Neurosci. (2017) 18:671–84. 10.1038/nrn.2017.112 28951611

[6] Leekam SR Nieto C Libby SJ Wing L Gould J. Describing the sensory abnormalities of children and adults with autism. J Autism Dev Disord. (2007) 37:894–910. 10.1007/s10803-006-0218-7 17016677

[7] Cascio C McGlone F Folger S Tannan V Baranek G Pelphrey KA. Tactile perception in adults with autism: a multidimensional psychophysical study. J Autism Dev Disord. (2008) 38:127–37. 10.1007/s10803-007-0370-8 17415630 PMC2185746

[8] Ben-Sasson A Hen L Fluss R Cermak SA Engel-Yeger B Gal E. meta-analysis of sensory modulation symptoms in individuals with autism spectrum disorders. J Autism Dev Disord. (2009) 39:1–11. 10.1007/s10803-008-0593-3 18512135

[9] Jones RSP Quigney C Huws JC. First-hand accounts of sensory perceptual experiences in autism: a qualitative analysis. J Intellect Dev Disabil. (2003) 28:112–21. 10.1080/1366825031000147058

[10] Hudac CM Friedman NR Ward VR Estreicher RE Dorsey GC Bernier RA. Characterizing sensory phenotypes of subgroups with a known genetic etiology pertaining to diagnoses of autism spectrum disorder and intellectual disability. J Autism Dev Disord. (2023)1–16. 10.1007/s10803-023-05897-9 37031308 PMC10083138

[11] Lyons-Warren AM McCormack MC Holder JL. Sensory processing phenotypes in phelan-McDermid syndrome and SYNGAP1-related intellectual disability. Brain Sci. (2022) 12:137. 10.3390/brainsci12020137 35203901 PMC8869824

[12] Brockevelt BL Nissen R Schweinle WE Kurtz E Larson KJ. A comparison of the sensory profile scores of children with autism and an age- and gender-matched sample. S D Med J S D State Med Assoc. (2013) 66:459–465. 24383262

[13] Simpson K Adams D Alston-Knox C Heussler HS Keen D. Exploring the sensory profiles of children on the autism spectrum using the short sensory profile-2 (SSP-2). J Autism Dev Disord. (2019) 49:2069–79. 10.1007/s10803-019-03889-2 30673910

[14] Uljarević M Lane A Kelly A Leekam S. Sensory subtypes and anxiety in older children and adolescents with autism spectrum disorder. Autism Res Off J Int Soc Autism Res. (2016) 9:1073–8. 10.1002/aur.1602 26765165

[15] Lyons-Warren AM Wangler MF Wan Y-W. Cluster analysis of short sensory profile data reveals sensory-based subgroups in autism spectrum disorder. Int J Mol Sci. (2022) 23:13030. 10.3390/ijms232113030 36361815 PMC9655407

[16] Blakemore S-J Tavassoli T Calò S Thomas RM Catmur C Frith U. Tactile sensitivity in Asperger syndrome. Brain Cogn. (2006) 61:5–13. 10.1016/j.bandc.2005.12.013 16500009

[17] Danesh AA Howery S Aazh H Kaf W Eshraghi AA. Hyperacusis in autism spectrum disorders. Audiol Res. (2021) 11:547–56. 10.3390/audiolres11040049 34698068 PMC8544234

[18] Rotschafer SE. Auditory discrimination in autism spectrum disorder. Front Neurosci. (2021) 15:651209. 10.3389/fnins.2021.651209 34211363 PMC8239241

[19] Rotschafer SE Razak KA. Auditory processing in fragile x syndrome. Front Cell Neurosci. (2014) 8:19. 10.3389/fncel.2014.00019 24550778 PMC3912505

[20] Williams ZJ He JL Cascio CJ Woynaroski TG. A review of decreased sound tolerance in autism: definitions, phenomenology, and potential mechanisms. Neurosci Biobehav Rev. (2021) 121:1–17. 10.1016/j.neubiorev.2020.11.030 33285160 PMC7855558

[21] Alcántara JI Weisblatt EJL Moore BCJ Bolton PF. Speech-in-noise perception in high-functioning individuals with autism or Asperger's syndrome. J Child Psychol Psychiatry. (2004) 45:1107–14. 10.1111/j.1469-7610.2004.t01-1-00303.x 15257667

[22] Groen WB van Orsouw L Huurne N Swinkels S van der Gaag R-J Buitelaar JK Zwiers MP. Intact spectral but abnormal temporal processing of auditory stimuli in autism. J Autism Dev Disord. (2009) 39:742–50. 10.1007/s10803-008-0682-3 19148738

[23] Foss-Feig JH Schauder KB Key AP Wallace MT Stone WL. Audition-specific temporal processing deficits associated with language function in children with autism spectrum disorder. Autism Res Off J Int Soc Autism Res. (2017) 10:1845–56. 10.1002/aur.1820 28632303 PMC6007978

[24] Plaisted K O'Riordan M Baron-Cohen S. Enhanced visual search for a conjunctive target in autism: a research note. J Child Psychol Psychiatry. (1998) 39:777–83. 9690940

[25] Orekhova EV Manyukhina VO Galuta IA Prokofyev AO Goiaeva DE Obukhova TS. Gamma oscillations point to the role of primary visual cortex in atypical motion processing in autism. PLoS ONE. (2023) 18:e0281531. 10.1371/journal.pone.0281531 36780507 PMC9925089

[26] Simmons DR Robertson AE McKay LS Toal E McAleer P Pollick FE. Vision in autism spectrum disorders. Vision Res. (2009) 49:2705–39. 10.1016/j.visres.2009.08.005 19682485

[27] Orefice LL. Peripheral somatosensory neuron dysfunction: emerging roles in autism spectrum disorders. Neuroscience. (2020) 445:120–9. 10.1016/j.neuroscience.2020.01.039 32035119 PMC7415509

[28] Green SA Rudie JD Colich NL Wood JJ Shirinyan D Hernandez L. Over reactive brain responses to sensory stimuli in youth with autism spectrum disorders. J Am Acad Child Adolesc Psychiatry. (2013) 52:1158–72. 10.1016/j.jaac.2013.08.004 24157390 PMC3820504

[29] Green SA Hernandez L Tottenham N Krasileva K Bookheimer SY Dapretto M. Neurobiology of sensory over responsivity in youth with autism spectrum disorders. JAMA Psychiatry. (2015) 72:778–86. 10.1001/jamapsychiatry.2015.0737 26061819 PMC4861140

[30] Gandal MJ Haney JR Wamsley B Yap CX Parhami S Emani PS. Broad transcriptomic dysregulation occurs across the cerebral cortex in ASD. Nature. (2022) 611:532–9. 10.1038/s41586-022-05377-7 36323788 PMC9668748

[31] Balasco L Provenzano G Bozzi Y. Sensory abnormalities in autism spectrum disorders: a focus on the tactile domain from genetic mouse models to the clinic. Front Psychiatry. (2020) 10:1016. 10.3389/fpsyt.2019.01016 32047448 PMC6997554

[32] Orefice LL Mosko JR Morency DT Wells MF Tasnim A Mozeika SM. Targeting peripheral somatosensory neurons to improve tactile-related phenotypes in ASD Models. Cell. (2019) 178:867–86. 10.1016/j.cell.2019.07.024 31398341 PMC6704376

[33] Balasco L Pagani M Pangrazzi L Chelini G Ciancone Chama AG Shlosman E. Abnormal whisker-dependent behaviors and altered cortico-hippocampal connectivity in shank3b-/- Mice. Cereb Cortex N Y N. (2022) 32:3042–56. 10.1093/cercor/bhab399 34791077 PMC9290535

[34] Michaelson SD Ozkan ED Aceti M Maity S Llamosas N Weldon M. SYNGAP1 heterozygosity disrupts sensory processing by reducing touch-related activity within somatosensory cortex circuits. Nat Neurosci. (2018) 21:1–13. 10.1038/s41593-018-0268-0 30455457 PMC6309426

[35] Orefice LL Zimmerman AL Chirila AM Sleboda SJ Head JP Ginty DD. Peripheral mechanosensory neuron dysfunction underlies tactile and behavioral deficits in mouse models of ASDs. Cell. (2016) 166:299–313. 10.1016/j.cell.2016.05.033 27293187 PMC5567792

[36] Chen Q Deister CA Gao X Guo B Lynn-Jones T Chen N. Dysfunction of cortical GABAergic neurons leads to sensory hyper-reactivity in a Shank3 mouse model of ASD. Nat Neurosci. (2020) 23:520–32. 10.1038/s41593-020-0598-6 32123378 PMC7131894

[37] He CX Cantu DA Mantri SS Zeiger WA Goel A Portera-Cailliau C. Tactile defensiveness and impaired adaptation of neuronal activity in the *Fmr1* knock-out mouse model of autism. J Neurosci. (2017) 37:6475–87. 10.1523/JNEUROSCI.0651-17.2017 28607173 PMC5511879

[38] Dawes JM Weir GA Middleton SJ Patel R Chisholm KI Pettingill P. Immune or genetic-mediated disruption of CASPR2 causes pain hypersensitivity due to enhanced primary afferent excitability. Neuron. (2018) 97:806-822.e10. 10.1016/j.neuron.2018.01.033 29429934 PMC6011627

[39] Ko H-G Oh S-B Zhuo M Kaang B-K. Reduced acute nociception and chronic pain in Shank2-/- mice. Mol Pain. (2016) 12:56. 10.1177/1744806916647056 27145803 PMC4956181

[40] Goel A Cantu DA Guilfoyle J Chaudhari GR Newadkar A Todisco B. Impaired perceptual learning in a mouse model of Fragile X syndrome is mediated by parvalbumin neuron dysfunction and is reversible. Nat Neurosci. (2018) 21:1404–11. 10.1038/s41593-018-0231-0 30250263 PMC6161491

[41] Del Rosario J Speed A Arrowood H Motz C Pardue M Haider B. Diminished cortical excitation and elevated inhibition during perceptual impairments in a mouse model of autism. Cereb Cortex N Y N. (2021) 31:3462–74. 10.1093/cercor/bhab025 33677512 PMC8525192

[42] Leach PT Crawley JN. Touchscreen learning deficits in Ube3a, Ts65Dn and Mecp2 mouse models of neurodevelopmental disorders with intellectual disabilities. Genes Brain Behav. (2018) 17:e12452. 10.1111/gbb.12452 29266714 PMC6013336

[43] Ash RT Palagina G Fernandez-Leon JA Park J Seilheimer R Lee S. Increased reliability of visually-evoked activity in area v1 of the mecp2-duplication mouse model of autism. J Neurosci Off J Soc Neurosci. (2022) 42:6469–82. 10.1523/JNEUROSCI.0654-22.2022 35831173 PMC9398540

[44] Yang C Tian Y Su F Wang Y Liu M Wang H. Restoration of FMRP expression in adult V1 neurons rescues visual deficits in a mouse model of fragile X syndrome. Protein Cell. (2022) 13:203–19. 10.1007/s13238-021-00878-z 34714519 PMC8901859

[45] Zhang D Yu B Liu J Jiang W Xie T Zhang R. Altered visual cortical processing in a mouse model of MECP2 duplication syndrome. Sci Rep. (2017) 7:6468. 10.1038/s41598-017-06916-3 28743991 PMC5526895

[46] Nielsen DM Derber WJ McClellan DA Crnic LS. Alterations in the auditory startle response in Fmr1 targeted mutant mouse models of fragile X syndrome. Brain Res. (2002) 927:8–17. 10.1016/s0006-8993(01)03309-1 11814427

[47] Rais M Binder DK Razak KA Ethell IM. Sensory processing phenotypes in fragile X syndrome. ASN Neuro. (2018) 10:1759091418801092. 10.1177/1759091418801092 30231625 PMC6149018

[48] Auerbach BD Manohar S Radziwon K Salvi R. Auditory hypersensitivity and processing deficits in a rat model of fragile X syndrome. Neurobiol Dis. (2021) 161:105541. 10.1016/j.nbd.2021.105541 34751141

[49] Rubenstein JLR Merzenich MM. Model of autism: increased ratio of excitation/inhibition in key neural systems. Genes Brain Behav. (2003) 2:255–67. 10.1034/j.1601-183x.2003.00037.x 14606691 PMC6748642

[50] Sohal VS Rubenstein JLR. Excitation-inhibition balance as a framework for investigating mechanisms in neuropsychiatric disorders. Mol Psychiatry. (2019) 24:1248–57. 10.1038/s41380-019-0426-0 31089192 PMC6742424

[51] Bolton PF Carcani-Rathwell I Hutton J Goode S Howlin P Rutter M. Epilepsy in autism: features and correlates. Br J Psychiatry J Ment Sci. (2011) 198:289–94. 10.1192/bjp.bp.109.076877 21972278 PMC3065774

[52] Markram K Markram H. The intense world theory – a unifying theory of the neurobiology of autism. Front Hum Neurosci. (2010) 4:224. 10.3389/fnhum.2010.00224 21191475 PMC3010743

[53] Antoine MW Langberg T Schnepel P Feldman DE. Increased excitation-inhibition ratio stabilizes synapse and circuit excitability in four autism mouse models. Neuron. (2019) 101:648–61. 10.1016/j.neuron.2018.12.026 30679017 PMC6733271

[54] Filice F Janickova L Henzi T Bilella A Schwaller B. The parvalbumin hypothesis of autism spectrum disorder. Front Cell Neurosci. (2020) 14:577525. 10.3389/fncel.2020.577525 33390904 PMC7775315

[55] Rudy B Fishell G Lee S Hjerling-Leffler J. Three groups of interneurons account for nearly 100% of neocortical GABAergic neurons. Dev Neurobiol. (2011) 71:45–61. 10.1002/dneu.20853 21154909 PMC3556905

[56] Ariza J Rogers H Hashemi E Noctor SC Martínez-Cerdeño V. The number of chandelier and basket cells are differentially decreased in prefrontal cortex in autism. Cereb Cortex N Y N. (2018) 28:411–20. 10.1093/cercor/bhw349 28122807 PMC6676950

[57] Hashemi E Ariza J Rogers H Noctor SC Martínez-Cerdeño V. The number of parvalbumin-expressing interneurons is decreased in the prefrontal cortex in autism. Cereb Cortex N Y N. (2017) 27:1931–43. 10.1093/cercor/bhw021 26922658 PMC6074948

[58] Contractor A Ethell IM Portera-Cailliau C. Cortical interneurons in autism. Nat Neurosci. (2021) 24:1648–59. 10.1038/s41593-021-00967-6 34848882 PMC9798607

[59] Nomura T. Interneuron dysfunction and inhibitory deficits in autism and fragile X syndrome. Cells. (2021) 10:2610. 10.3390/cells10102610 34685590 PMC8534049

[60] Reh RK Dias BG Nelson CA Kaufer D Werker JF Kolb B. Critical period regulation across multiple timescales. Proc Natl Acad Sci U S A. (2020) 117:23242–51. 10.1073/pnas.1820836117 32503914 PMC7519216

[61] Kourdougli N Suresh A Liu B Juarez P Lin A Chung DT. Improvement of sensory deficits in fragile X mice by increasing cortical interneuron activity after the critical period. Neuron. (2023) 25:S0896–6273. 10.1016/j.neuron.2023.06.009 37451263 PMC10529373

[62] Nelson SB Valakh V. Excitatory/inhibitory balance and circuit homeostasis in autism spectrum disorders. Neuron. (2015) 87:684–98. 10.1016/j.neuron.2015.07.033 26291155 PMC4567857

[63] Turrigiano G. Homeostatic synaptic plasticity: local and global mechanisms for stabilizing neuronal function. Cold Spring Harb Perspect Biol. (2012) 4:a005736. 10.1101/cshperspect.a005736 22086977 PMC3249629

[64] Mullins C Fishell G Tsien RW. Unifying views of autism spectrum disorders: a consideration of auto regulatory feedback loops. Neuron. (2016) 89:1131–56. 10.1016/j.neuron.2016.02.017 26985722 PMC5757244

[65] Genç Ö An J-Y Fetter RD Kulik Y Zunino G Sanders SJ. Homeostatic plasticity fails at the intersection of autism-gene mutations and a novel class of common genetic modifiers. eLife. (2020) 9:775. 10.7554/eLife.55775 32609087 PMC7394548

[66] Monday HR Younts TJ Castillo PE. Long-term plasticity of neurotransmitter release: emerging mechanisms and contributions to brain function and disease. Annu Rev Neurosci. (2018) 41:299–322. 10.1146/annurev-neuro-080317-062155 29709205 PMC6238218

[67] Bourgeron T. From the genetic architecture to synaptic plasticity in autism spectrum disorder. Nat Rev Neurosci. (2015) 16:551–63. 10.1038/nrn3992 26289574

[68] Bear MF Huber KM Warren ST. The mGluR theory of fragile X mental retardation. Trends Neurosci. (2004) 27:370–7. 10.1016/j.tins.2004.04.009 15219735

[69] Huber KM Gallagher SM Warren ST Bear MF. Altered synaptic plasticity in a mouse model of fragile X mental retardation. Proc Natl Acad Sci U S A. (2002) 99:7746–50. 10.1073/pnas.122205699 12032354 PMC124340

[70] Bateup HS Johnson CA Denefrio CL Saulnier JL Kornacker K Sabatini BL. Excitatory/inhibitory synaptic imbalance leads to hippocampal hyperexcitability in mouse models of tuberous sclerosis. Neuron. (2013) 78:510–22. 10.1016/j.neuron.2013.03.017 23664616 PMC3690324

[71] Dang R Qi J Liu A Ren Q Lv D Han L. Regulation of hippocampal long term depression by Neuroligin 1. Neuropharmacology. (2018) 143:205–16. 10.1016/j.neuropharm.2018.09.035 30266599

[72] Lee K Vyas Y Garner CC Montgomery JM. Autism-associated Shank3 mutations alter mGluR expression and mGluR-dependent but not NMDA receptor-dependent long-term depression. Synap N Y N. (2019) 73:e22097. 10.1002/syn.22097 30868621

[73] Auerbach BD Osterweil EK Bear MF. Mutations causing syndromic autism define an axis of synaptic pathophysiology. Nature. (2011) 480:63–8. 10.1038/nature10658 22113615 PMC3228874

[74] Klein ME Monday H Jordan BA. Proteostasis and RNA binding proteins in synaptic plasticity and in the pathogenesis of neuropsychiatric disorders. Neural Plast. (2016) 2016:3857934. 10.1155/2016/3857934 26904297 PMC4745388

[75] Santini E Klann E. Reciprocal signaling between translational control pathways and synaptic proteins in autism spectrum disorders. Sci Signal. (2014) 7:re10. 10.1126/scisignal.2005832 25351249 PMC6002803

[76] Bagni C Zukin RS A. Synaptic perspective of fragile X syndrome and autism spectrum disorders. Neuron. (2019) 101:1070–88. 10.1016/j.neuron.2019.02.041 30897358 PMC9628679

[77] Forrest MP Parnell E Penzes P. Dendritic structural plasticity and neuropsychiatric disease. Nat Rev Neurosci. (2018) 19:215–34. 10.1038/nrn.2018.16 29545546 PMC6442683

[78] Zieger HL Choquet D. Nanoscale synapse organization and dysfunction in neurodevelopmental disorders. Neurobiol Dis. (2021) 158:105453. 10.1016/j.nbd.2021.105453 34314857

[79] LeBlanc JJ Fagiolini M. Autism: a “critical period” disorder? Neural Plast. (2011) 2011:921680. 10.1155/2011/921680 21826280 PMC3150222

[80] Rupert DD Shea SD. Parvalbumin-Positive Interneurons Regulate Cortical Sensory Plasticity in Adulthood and Development Through Shared Mechanisms. Front Neural Circuits. (2022) 16:886629. 10.3389/fncir.2022.886629 35601529 PMC9120417

[81] Varcin KJ Jeste SS. The emergence of autism spectrum disorder (ASD): insights gained from studies of brain and behaviour in high-risk infants. Curr Opin Psychiatry. (2017) 30:85–91. 10.1097/YCO.0000000000000312 28009726 PMC5915621

[82] Aceti M Creson TK Vaissiere T Rojas C Huang W-C Wang Y-X. Syngap1 haploin sufficiency damages a postnatal critical period of pyramidal cell structural maturation linked to cortical circuit assembly. Biol Psychiatry. (2015) 77:805–15. 10.1016/j.biopsych.2014.08.001 25444158 PMC4326604

[83] Allegra M Genovesi S Maggia M Cenni MC Zunino G Sgadò P. Altered GABAergic markers, increased binocularity and reduced plasticity in the visual cortex of Engrailed-2 knockout mice. Front Cell Neurosci. (2014) 8:163. 10.3389/fncel.2014.00163 24987331 PMC4060086

[84] Yashiro K Riday TT Condon KH Roberts AC Bernardo DR Prakash R. Ube3a is required for experience-dependent maturation of the neocortex. Nat Neurosci. (2009) 12:777–83. 10.1038/nn.2327 19430469 PMC2741303

[85] Krishnan K Wang B-S Lu J Wang L Maffei A Cang J. MeCP2 regulates the timing of critical period plasticity that shapes functional connectivity in primary visual cortex. Proc Natl Acad Sci U S A. (2015) 112:E4782–4791. 10.1073/pnas.1506499112 26261347 PMC4553776

[86] Bureau I Shepherd GMG Svoboda K. Circuit and plasticity defects in the developing somatosensory cortex of Fmr1 knock-out mice. J Neurosci. (2008) 28:5178–88. 10.1523/JNEUROSCI.1076-08.2008 18480274 PMC2696604

[87] Harlow EG Till SM Russell TA Wijetunge LS Kind P Contractor A. Critical period plasticity is disrupted in the barrel cortex of FMR1 knockout mice. Neuron. (2010) 65:385–98. 10.1016/j.neuron.2010.01.024 20159451 PMC2825250

[88] He L Liu N Cheng T Chen X Li Y Shu Y. Conditional deletion of Mecp2 in parvalbumin-expressing GABAergic cells results in the absence of critical period plasticity. Nat Commun. (2014) 5:5036. 10.1038/ncomms6036 25297674

[89] Nomura T Musial TF Marshall JJ Zhu Y Remmers CL Xu J. Delayed maturation of fast-spiking interneurons is rectified by activation of the TrkB receptor in the mouse model of fragile X syndrome. J Neurosci. (2017) 37:11298–310. 10.1523/JNEUROSCI.2893-16.2017 29038238 PMC5700416

[90] Song YJ Xing B Barbour AJ Zhou C Jensen FE. Dysregulation of GABAA receptor-mediated neurotransmission during the auditory cortex critical period in the fragile X syndrome mouse model. Cereb Cortex. (2022) 32:197–215. 10.1093/cercor/bhab203 34223875 PMC8634585

[91] Dani VS Chang Q Maffei A Turrigiano GG Jaenisch R Nelson SB. Reduced cortical activity due to a shift in the balance between excitation and inhibition in a mouse model of Rett Syndrome. Proc Natl Acad Sci. (2005) 102:12560–5. 10.1073/pnas.0506071102 16116096 PMC1194957

[92] Nakai N Nagano M Saitow F Watanabe Y Kawamura Y Kawamoto A. Serotonin rebalances cortical tuning and behavior linked to autism symptoms in 15q11-13 CNV mice. Sci Adv. (2017) 3:e1603001. 10.1126/sciadv.1603001 28691086 PMC5479676

[93] Banerjee A Rikhye RV Breton-Provencher V Tang X Li C Li K. Jointly reduced inhibition and excitation underlies circuit-wide changes in cortical processing in Rett syndrome. Proc Natl Acad Sci U S A. (2016) 113:E7287–96. 10.1073/pnas.1615330113 27803317 PMC5135376

[94] Rotschafer S Razak K. Altered auditory processing in a mouse model of fragile X syndrome. Brain Res. (2013) 1506:12–24. 10.1016/j.brainres.2013.02.038 23458504

[95] Wadle SL Schmitt TTX Engel J Kurt S Hirtz JJ. Altered population activity and local tuning heterogeneity in auditory cortex of Cacna2d3-deficient mice. Biol Chem. (2023) 404:607–17. 10.1515/hsz-2022-0269 36342370

[96] Anomal RF de Villers-Sidani E Brandão JA Diniz R Costa MR Romcy-Pereira RN. Impaired processing in the primary auditory cortex of an animal model of autism. Front Syst Neurosci. (2015) 9:158. 10.3389/fnsys.2015.00158 26635548 PMC4644803

[97] Juczewski K von Richthofen H Bagni C Celikel T Fisone G Krieger P. Somatosensory map expansion and altered processing of tactile inputs in a mouse model of fragile X syndrome. Neurobiol Dis. (2016) 96:201–15. 10.1016/j.nbd.2016.09.007 27616423

[98] Zhou C Yan S Qian S Wang Z Shi Z Xiong Y. Atypical response properties of the auditory cortex of awake MECP2-overexpressing mice. Front Neurosci. (2019) 13:439. 10.3389/fnins.2019.00439 31133783 PMC6515258

[99] Wallace ML van Woerden GM Elgersma Y Smith SL Philpot BD. Ube3a loss increases excitability and blunts orientation tuning in the visual cortex of Angelman syndrome model mice. J Neurophysiol. (2017) 118:634–46. 10.1152/jn.00618.2016 28468997 PMC5511875

[100] Cheyne JE Zabouri N Baddeley D Lohmann C. Spontaneous activity patterns are altered in the developing visual cortex of the Fmr1 knockout mouse. Front Neural Circuits. (2019) 13:57. 10.3389/fncir.2019.00057 31616256 PMC6775252

[101] Ethridge LE White SP Mosconi MW Wang J Pedapati EV Erickson CA. Neural synchronization deficits linked to cortical hyper-excitability and auditory hypersensitivity in fragile X syndrome. Mol Autism. (2017) 8:22. 10.1186/s13229-017-0140-1 28596820 PMC5463459

[102] Gonçalves JT Anstey JE Golshani P Portera-Cailliau C. Circuit level defects in the developing neocortex of Fragile X mice. Nat Neurosci. (2013) 16:903–9. 10.1038/nn.3415 23727819 PMC3695061

[103] Hays SA Huber KM Gibson JR. Altered neocortical rhythmic activity states in Fmr1 KO Mice Are Due to enhanced mGluR5 signaling and involve changes in excitatory circuitry. J Neurosci. (2011) 31:14223–34. 10.1523/JNEUROSCI.3157-11.2011 21976507 PMC3207280

[104] Lazaro MT Taxidis J Shuman T Bachmutsky I Ikrar T Santos R. Reduced prefrontal synaptic connectivity and disturbed oscillatory population dynamics in the CNTNAP2 model of autism. Cell Rep. (2019) 27:2567–78. 10.1016/j.celrep.2019.05.006 31141683 PMC6553483

[105] O'Donnell C Gonçalves JT Portera-Cailliau C Sejnowski TJ. Beyond excitation/inhibition imbalance in multidimensional models of neural circuit changes in brain disorders. Elife. (2017) 6:e26724. 10.7554/eLife.26724 29019321 PMC5663477

[106] He JL Wodka E Tommerdahl M Edden RAE Mikkelsen M Mostofsky SH. Disorder-specific alterations of tactile sensitivity in neurodevelopmental disorders. Commun Biol. (2021) 4:1–15. 10.1038/s42003-020-01592-y 33483581 PMC7822903

[107] Puts NAJ Wodka EL Tommerdahl M Mostofsky SH Edden RAE. Impaired tactile processing in children with autism spectrum disorder. J Neurophysiol. (2014) 111:1803–11. 10.1152/jn.00890.2013 24523518 PMC4044368

[108] Schelinski S von Kriegstein K. Brief report: speech-in-noise recognition and the relation to vocal pitch perception in adults with autism spectrum disorder and typical development. J Autism Dev Disord. (2020) 50:356–63. 10.1007/s10803-019-04244-1 31583624

[109] Dinstein I Heeger DJ Lorenzi L Minshew NJ Malach R Behrmann M. Unreliable evoked responses in autism. Neuron. (2012) 75:981–91. 10.1016/j.neuron.2012.07.026 22998867 PMC3457023

[110] Coskun MA Varghese L Reddoch S Castillo EM Pearson DA Loveland KA. How somatic cortical maps differ in autistic and typical brains. Neuroreport. (2009) 20:175–9. 10.1097/WNR.0b013e32831f47d1 19057419

[111] Frey H-P Molholm S Lalor EC Russo NN Foxe JJ. Atypical cortical representation of peripheral visual space in children with an autism spectrum disorder. Eur J Neurosci. (2013) 38:2125–38. 10.1111/ejn.12243 23692590 PMC4587666

[112] Smith H Lane C Al-Jawahiri R. Freeth M. Sensory processing in 16p112 deletion and 16p112 duplication Autism. Res Off J Int Soc Autism Res. (2022) 15:2081–98. 10.1002/aur.2802 36053814 PMC9826336

[113] Guo B Chen J Chen Q Ren K Feng D Mao H. Anterior cingulate cortex dysfunction underlies social deficits in Shank3 mutant mice. Nat Neurosci. (2019) 22:1223–34. 10.1038/s41593-019-0445-9 31332372

[114] Zhang Y Bonnan A Bony G Ferezou I Pietropaolo S Ginger M. Dendritic channelopathies contribute to neocortical and sensory hyperexcitability in Fmr1(-/y) mice. Nat Neurosci. (2014) 17:1701–9. 10.1038/nn.3864 25383903

[115] Millin R Kolodny T Flevaris AV Kale AM Schallmo M-P Gerdts J. Reduced auditory cortical adaptation in autism spectrum disorder. Elife. (2018) 7:e36493. 10.7554/eLife.36493 30362457 PMC6203433

[116] Tannan V Holden JK Zhang Z Baranek GT Tommerdahl MA. Perceptual metrics of individuals with autism provide evidence for disinhibition. Autism Res. (2008) 1:223–30. 10.1002/aur.34 19360672 PMC3340566

[117] Lovelace JW Wen TH Reinhard S Hsu MS Sidhu H Ethell IM. Matrix metalloproteinase-9 deletion rescues auditory evoked potential habituation deficit in a mouse model of Fragile X Syndrome. Neurobiol Dis. (2016) 89:126–35. 10.1016/j.nbd.2016.02.002 26850918 PMC4785038

[118] Wallace ML Burette AC Weinberg RJ Philpot BD. Maternal loss of Ube3a produces an excitatory/inhibitory imbalance through neuron type-specific synaptic defects. Neuron. (2012) 74:793–800. 10.1016/j.neuron.2012.03.036 22681684 PMC3372864

[119] Buzsáki G Draguhn A. Neuronal oscillations in cortical networks. Science. (2004) 304:1926–9. 10.1126/science.1099745 15218136

[120] Ai L Ro T. The phase of prestimulus alpha oscillations affects tactile perception. J Neurophysiol. (2014) 111:1300–7. 10.1152/jn.00125.2013 24381028

[121] Busch NA Dubois J Van Rullen R. The phase of ongoing EEG oscillations predicts visual perception. J Neurosci. (2009) 29:7869–76. 10.1523/JNEUROSCI.0113-09.2009 19535598 PMC6665641

[122] Gruber WR Klimesch W Sauseng P Doppelmayr M. Alpha phase synchronization predicts P1 and N1 latency and amplitude size. Cereb Cortex N Y N. (2005) 15:371–7. 10.1093/cercor/bhh139 15749980

[123] Iemi L Chaumon M Crouzet SM Busch NA. Spontaneous neural oscillations bias perception by modulating baseline excitability. J Neurosci Off J Soc Neurosci. (2017) 37:807–19. 10.1523/JNEUROSCI.1432-16.2016 28123017 PMC6597018

[124] Samaha J Iemi L Haegens S Busch NA. Spontaneous brain oscillations and perceptual decision-making. Trends Cogn Sci. (2020) 24:639–53. 10.1016/j.tics.2020.05.004 32513573

[125] Strauß A Henry MJ Scharinger M Obleser J. Alpha phase determines successful lexical decision in noise. J Neurosci Off J Soc Neurosci. (2015) 35:3256–62. 10.1523/JNEUROSCI.3357-14.2015 25698760 PMC6605582

[126] Simon DM Wallace MT. Dysfunction of sensory oscillations in autism spectrum disorder. Neurosci Biobehav Rev. (2016) 68:848–61. 10.1016/j.neubiorev.2016.07.016 27451342 PMC5119455

[127] Edgar JC Khan SY Blaskey L Chow VY Rey M Gaetz W. Neuro magnetic oscillations predict evoked-response latency delays and core language deficits in autism spectrum disorders. J Autism Dev Disord. (2015) 45:395–405. 10.1007/s10803-013-1904-x 23963591 PMC5012005

[128] Khan S Michmizos K Tommerdahl M Ganesan S Kitzbichler MG Zetino M. Somatosensory cortex functional connectivity abnormalities in autism show opposite trends, depending on direction and spatial scale. Brain J Neurol. (2015) 138:1394–409. 10.1093/brain/awv043 25765326 PMC5013931

[129] Milne E Scope A Pascalis O Buckley D Makeig S. Independent component analysis reveals atypical electroencephalographic activity during visual perception in individuals with autism. Biol Psychiatry. (2009) 65:22–30. 10.1016/j.biopsych.2008.07.017 18774554

[130] Rojas DC Maharajh K Teale P Rogers SJ. Reduced neural synchronization of gamma-band MEG oscillations in first-degree relatives of children with autism. BMC Psychiatry. (2008) 8:66. 10.1186/1471-244X-8-66 18673566 PMC2518921

[131] Snijders TM Milivojevic B Kemner C. Atypical excitation-inhibition balance in autism captured by the gamma response to contextual modulation. NeuroImage Clin. (2013) 3:65–72. 10.1016/j.nicl.2013.06.015 24179850 PMC3791282

[132] Stroganova TA Orekhova EV Prokofyev AO Tsetlin MM Gratchev VV Morozov AA. High-frequency oscillatory response to illusory contour in typically developing boys and boys with autism spectrum disorders. Cortex J Devoted Study Nerv Syst Behav. (2012) 48:701–17. 10.1016/j.cortex.2011.02.016 21458787

[133] Sun L Grützner C Bölte S Wibral M Tozman T Schlitt S. Impaired gamma-band activity during perceptual organization in adults with autism spectrum disorders: evidence for dysfunctional network activity in frontal-posterior cortices. J Neurosci Off J Soc Neurosci. (2012) 32:9563–73. 10.1523/JNEUROSCI.1073-12.2012 22787042 PMC6622268

[134] Wilson TW Rojas DC Reite ML Teale PD Rogers SJ. Children and adolescents with autism exhibit reduced MEG steady-state gamma responses. Biol Psychiatry. (2007) 62:192–7. 10.1016/j.biopsych.2006.07.002 16950225 PMC2692734

[135] Brown C Gruber T Boucher J Rippon G Brock J. Gamma abnormalities during perception of illusory figures in autism. Cortex J Devoted Study Nerv Syst Behav. (2005) 41:364–76. 10.1016/s0010-9452(08)70273-9 15871601

[136] Buard I Rogers SJ Hepburn S Kronberg E Rojas DC. Altered oscillation patterns and connectivity during picture naming in autism. Front Hum Neurosci. (2013) 7:742. 10.3389/fnhum.2013.00742 24265611 PMC3821038

[137] Côté V Knoth IS Agbogba K Vannasing P Côté L Major P. Differential auditory brain response abnormalities in two intellectual disability conditions: SYNGAP1 mutations and Down syndrome. Clin Neurophysiol. (2021) 132:1802–12. 10.1016/j.clinph.2021.03.054 34130248

[138] Gandal MJ Edgar JC Ehrlichman RS Mehta M Roberts TPL Siegel SJ. Validating γ oscillations and delayed auditory responses as translational biomarkers of autism. Biol Psychiatry. (2010) 68:1100–6. 10.1016/j.biopsych.2010.09.031 21130222 PMC5070466

[139] David N Schneider TR Peiker I Al-Jawahiri R Engel AK Milne E. Variability of cortical oscillation patterns: a possible endophenotype in autism spectrum disorders? Neurosci Biobehav Rev. (2016) 71:590–600. 10.1016/j.neubiorev.2016.09.031 27746319

[140] Murphy JW Foxe JJ Peters JB Molholm S. Susceptibility to distraction in autism spectrum disorder: probing the integrity of oscillatory alpha-band suppression mechanisms. Autism Res Off J Int Soc Autism Res. (2014) 7:442–58. 10.1002/aur.1374 24678054 PMC4183200

[141] Isler JR Martien KM Grieve PG Stark RI Herbert MR. Reduced functional connectivity in visual evoked potentials in children with autism spectrum disorder. Clin Neurophysiol Off J Int Fed Clin Neurophysiol. (2010) 121:2035–43. 10.1016/j.clinph.2010.05.004 20605520

[142] Lazarev VV Pontes A Mitrofanov AA deAzevedo LC. Reduced interhemispheric connectivity in childhood autism detected by electroencephalographic photic driving coherence. J Autism Dev Disord. (2015) 45:537–47. 10.1007/s10803-013-1959-8 24097142

[143] Peiker I David N Schneider TR Nolte G Schöttle D Engel AK. Perceptual integration deficits in autism spectrum disorders are associated with reduced interhemispheric gamma-band coherence. J Neurosci Off J Soc Neurosci. (2015) 35:16352–61. 10.1523/JNEUROSCI.1442-15.2015 26674862 PMC6605515

[144] Jonak CR Lovelace JW Ethell IM Razak KA Binder DK. Multielectrode array analysis of EEG biomarkers in a mouse model of Fragile X Syndrome. Neurobiol Dis. (2020) 138:104794. 10.1016/j.nbd.2020.104794 32036032 PMC9038039

[145] Lovelace JW Ethell IM Binder DK Razak KA. Translation-relevant EEG phenotypes in a mouse model of Fragile X Syndrome. Neurobiol Dis. (2018) 115:39–48. 10.1016/j.nbd.2018.03.012 29605426 PMC5969806

[146] Sinclair D Featherstone R Naschek M Nam J Du A Wright S. GABA-B agonist baclofen normalizes auditory-evoked neural oscillations and behavioral deficits in the fmr1 knockout mouse model of fragile X syndrome. eNeuro. (2017) 4: 1–7. 10.1523/ENEURO.0380-16.2017 28451631 PMC5394929

[147] Janz P Bainier M Marashli S Schoenenberger P Valencia M Redondo RL. Neurexin1α knockout rats display oscillatory abnormalities and sensory processing deficits back-translating key endophenotypes of psychiatric disorders. Transl Psychiatry. (2022) 12:455. 10.1038/s41398-022-02224-1 36307390 PMC9616904

[148] Han S Tai C Westenbroek RE Yu FH Cheah CS Potter GB. Autistic-like behaviour in Scn1a+/– mice and rescue by enhanced GABA-mediated neurotransmission. Nature. (2012) 489:385–90. 10.1038/nature11356 22914087 PMC3448848

[149] Tai C Abe Y Westenbroek RE Scheuer T Catterall WA. Impaired excitability of somatostatin- and parvalbumin-expressing cortical interneurons in a mouse model of Dravet syndrome. Proc Natl Acad Sci. (2014) 111:E3139–48. 10.1073/pnas.1411131111 25024183 PMC4121787

[150] Favero M Sotuyo NP Lopez E Kearney JA Goldberg EM A. Transient developmental window of fast-spiking interneuron dysfunction in a mouse model of dravet syndrome. J Neurosci. (2018) 38:7912–27. 10.1523/JNEUROSCI.0193-18.2018 30104343 PMC6125809

[151] Tran CH Vaiana M Nakuci J Somarowthu A Goff KM Goldstein N. Interneuron desynchronization precedes seizures in a mouse model of Dravet syndrome. J Neurosci Off J Soc Neurosci. (2020) 40:2764–75. 10.1523/JNEUROSCI.2370-19.2020 32102923 PMC7096149

[152] Ogiwara I Miyamoto H Morita N Atapour N Mazaki E Inoue I. Nav11 localizes to axons of parvalbumin-positive inhibitory interneurons: a circuit basis for epileptic seizures in mice carrying an Scn1a gene mutation. J Neurosci. (2007) 27:5903–14. 10.1523/JNEUROSCI.5270-06.2007 17537961 PMC6672241

[153] Peixoto RT Wang W Croney DM Kozorovitskiy Y Sabatini BL. Early hyperactivity and precocious maturation of corticostriatal circuits in Shank3B–/– mice. Nat Neurosci. (2016) 19:716–24. 10.1038/nn.4260 26928064 PMC4846490

[154] Pagano J Landi S Stefanoni A Nardi G Albanesi M Bauer HF. Shank3 deletion in PV neurons is associated with abnormal behaviors and neuronal functions that are rescued by increasing GABAergic signaling. Mol Autism. (2023) 14:28. 10.1186/s13229-023-00557-2 37528484 PMC10394945

[155] Gonzalez D Tomasek M Hays S Sridhar V Ammanuel S Chang C-W. Audiogenic seizures in the Fmr1 knock-out mouse are induced by fmr1 deletion in subcortical, VGlut2-expressing excitatory neurons and require deletion in the inferior colliculus. J Neurosci Off J Soc Neurosci. (2019) 39:9852–63. 10.1523/JNEUROSCI.0886-19.2019 31666356 PMC6891051

[156] Gibson JR Bartley AF Hays SA Huber KM. Imbalance of neocortical excitation and inhibition and altered UP states reflect network hyperexcitability in the mouse model of fragile X syndrome. J Neurophysiol. (2008) 100:2615–26. 10.1152/jn.90752.2008 18784272 PMC2585391

[157] Arnett MT Herman DH McGee AW. Deficits in tactile learning in a mouse model of fragile X syndrome. PLoS ONE. (2014) 9:e109116. 10.1371/journal.pone.0109116 25296296 PMC4189789

[158] Balasco L Pagani M Pangrazzi L Chelini G Viscido F Chama AGC. Somatosensory cortex hyperconnectivity and impaired whisker-dependent responses in Cntnap2–/– mice. Neurobiol Dis. (2022) 169:105742. 10.1016/j.nbd.2022.105742 35483565

[159] Scott KE Mann RS Schormans AL Schmid S Allman BL. Hyperexcitable and immature-like neuronal activity in the auditory cortex of adult rats lacking the language-linked CNTNAP2 gene. Cereb Cortex N Y N. (2022) 32:4797–817. 10.1093/cercor/bhab517 35106542 PMC9626820

[160] Scott KE Schormans AL Pacoli KY De Oliveira C Allman BL Schmid S. Altered auditory processing, filtering, and reactivity in the cntnap2 knock-out rat model for neurodevelopmental disorders. J Neurosci Off J Soc Neurosci. (2018) 38:8588–604. 10.1523/JNEUROSCI.0759-18.2018 30126973 PMC6596223

[161] Krishnan K Lau BYB Ewall G Huang ZJ Shea SD. MECP2 regulates cortical plasticity underlying a learned behaviour in adult female mice. Nat Commun. (2017) 8:14077. 10.1038/ncomms14077 28098153 PMC5253927

[162] Morello N Schina R Pilotto F Phillips M Melani R Plicato O. Loss of Mecp2 causes atypical synaptic and molecular plasticity of parvalbumin-expressing interneurons reflecting rett syndrome-like sensorimotor defects. eNeuro. (2018) 5:2018. 10.1523/ENEURO.0086-18.2018 30255129 PMC6153339

[163] Durand S Patrizi A Quast KB Hachigian L Pavlyuk R Saxena A. Receptor regulation prevents regression of visual cortical function in the absence of Mecp2. Neuron. (2012) 76:1078–90. 10.1016/j.neuron.2012.12.004 23259945 PMC3733788

[164] Engineer CT Rahebi KC Borland MS Buell EP Centanni TM Fink MK. Degraded neural and behavioral processing of speech sounds in a rat model of Rett syndrome. Neurobiol Dis. (2015) 83:26–34. 10.1016/j.nbd.2015.08.019 26321676 PMC4674323

[165] Ramocki MB Zoghbi HY. Failure of neuronal homeostasis results in common neuropsychiatric phenotypes. Nature. (2008) 455:912–8. 10.1038/nature07457 18923513 PMC2696622

[166] Neklyudova A Smirnov K Rebreikina A Martynova O Sysoeva O. Electrophysiological and behavioral evidence for hyper- and hyposensitivity in rare genetic syndromes associated with autism. Genes. (2022) 13:671. 10.3390/genes13040671 35456477 PMC9027402

[167] Donato F Rompani SB Caroni P. Parvalbumin-expressing basket-cell network plasticity induced by experience regulates adult learning. Nature. (2013) 504:272–6. 10.1038/nature12866 24336286

[168] Selby L Zhang C Sun Q-Q. Major defects in neocortical GABAergic inhibitory circuits in mice lacking the fragile X mental retardation protein. Neurosci Lett. (2007) 412:227–32. 10.1016/j.neulet.2006.11.062 17197085 PMC1839948

[169] Wen TH Afroz S Reinhard SM Palacios AR Tapia K Binder DK. Genetic Reduction of matrix metalloproteinase-9 promotes formation of perineuronal nets around parvalbumin-expressing interneurons and normalizes auditory cortex responses in developing fmr1 knock-out mice. Cereb Cortex. (2018) 28:3951–64. 10.1093/cercor/bhx258 29040407 PMC6188540

[170] Filice F Vörckel KJ Sungur AÖ Wöhr M Schwaller B. Reduction in parvalbumin expression not loss of the parvalbumin-expressing GABA interneuron subpopulation in genetic parvalbumin and shank mouse models of autism. Mol Brain. (2016) 9:10. 10.1186/s13041-016-0192-8 26819149 PMC4729132

[171] Gogolla N Leblanc JJ Quast KB Südhof TC Fagiolini M Hensch TK. Common circuit defect of excitatory-inhibitory balance in mouse models of autism. J Neurodev Disord. (2009) 1:172–81. 10.1007/s11689-009-9023-x 20664807 PMC2906812

[172] Jung E-M Moffat JJ Liu J Dravid SM Gurumurthy CB Kim W-Y. Arid1b haploinsufficiency disrupts cortical interneuron development and mouse behavior. Nat Neurosci. (2017) 20:1694–707. 10.1038/s41593-017-0013-0 29184203 PMC5726525

[173] Peñagarikano O Abrahams BS Herman EI Winden KD Gdalyahu A Dong H. Absence of CNTNAP2 leads to epilepsy, neuronal migration abnormalities, and core autism-related deficits. Cell. (2011) 147:235–46. 10.1016/j.cell.2011.08.040 21962519 PMC3390029

[174] Vogt D Cho KKA Shelton SM Paul A Huang ZJ Sohal VS. Mouse Cntnap2 and human CNTNAP2 ASD alleles cell autonomously regulate PV+ cortical interneurons. Cereb Cortex. (2018) 28:3868–79. 10.1093/cercor/bhx248 29028946 PMC6455910

[175] Lauber E Filice F Schwaller B. Dysregulation of parvalbumin expression in the Cntnap2-/- mouse model of autism spectrum disorder. Front Mol Neurosci. (2018) 11:262. 10.3389/fnmol.2018.00262 30116174 PMC6082962

[176] Scott R Sánchez-Aguilera A van Elst K Lim L Dehorter N Bae SE. Loss of Cntnap2 causes axonal excitability deficits, developmental delay in cortical myelination, and abnormal stereotyped motor behavior. Cereb Cortex. (2019) 29:586–97. 10.1093/cercor/bhx341 29300891

[177] Deemyad T Puig S Papale AE Qi H LaRocca GM Aravind D. Lateralized decrease of Parvalbumin+ cells in the somatosensory cortex of ASD models is correlated with unilateral tactile hypersensitivity. Cereb Cortex N Y N. (2022) 32:554–68. 10.1093/cercor/bhab233 34347040 PMC8805834

[178] Bernard C Exposito-Alonso D Selten M Sanalidou S Hanusz-Godoy A Aguilera A. Cortical wiring by synapse type–specific control of local protein synthesis. Science. (2022) 378:eabm7466. 10.1126/science.abm7466 36423280 PMC7618116

[179] He Q Arroyo ED Smukowski SN Xu J Piochon C Savas JN. Critical period inhibition of NKCC1 rectifies synapse plasticity in the somatosensory cortex and restores adult tactile response maps in fragile X mice. Mol Psychiatry. (2019) 24:1732–47. 10.1038/s41380-018-0048-y 29703945 PMC6204122

[180] Lovelace JW Rais M Palacios AR Shuai XS Bishay S. Deletion of Fmr1 from forebrain excitatory neurons triggers abnormal cellular, EEG, and Behavioral Phenotypes in the auditory cortex of a mouse model of fragile X syndrome. Cereb Cortex N Y N. (2020) 30:969–88. 10.1093/cercor/bhz141 31364704 PMC7132927

[181] Filice F Lauber E Vörckel KJ Wöhr M Schwaller B. 17-β estradiol increases parvalbumin levels in Pvalb heterozygous mice and attenuates behavioral phenotypes with relevance to autism core symptoms. Mol Autism. (2018) 9:15. 10.1186/s13229-018-0199-3 29507711 PMC5833085

[182] Wöhr M Orduz D Gregory P Moreno H Khan U Vörckel KJ. Lack of parvalbumin in mice leads to behavioral deficits relevant to all human autism core symptoms and related neural morphofunctional abnormalities. Transl Psychiatry. (2015) 5:e525. 10.1038/tp.2015.19 25756808 PMC4354349

[183] Chao H-T Chen H Samaco RC Xue M Chahrour M Yoo J. Dysfunction in GABA signalling mediates autism-like stereotypies and Rett syndrome phenotypes. Nature. (2010) 468:263–9. 10.1038/nature09582 21068835 PMC3057962

[184] Zhang W Peterson M Beyer B Frankel WN Zhang Z. Loss of MeCP2 from forebrain excitatory neurons leads to cortical hyperexcitation and seizures. J Neurosci. (2014) 34:2754–63. 10.1523/JNEUROSCI.4900-12.2014 24523563 PMC3921436

[185] Ito-Ishida A Ure K Chen H Swann JW Zoghbi HY. Loss of MeCP2 in Parvalbumin-and somatostatin-expressing neurons in mice leads to distinct rett syndrome-like phenotypes. Neuron. (2015) 88:651–8. 10.1016/j.neuron.2015.10.029 26590342 PMC4656196

[186] Morin-Parent F Champigny C Lacroix A Corbin F Lepage J-F. Hyperexcitability and impaired intracortical inhibition in patients with fragile-X syndrome. Transl Psychiatry. (2019) 9:1–9. 10.1038/s41398-019-0650-z 31748507 PMC6868148

[187] Canitano R Palumbi R. Excitation/inhibition modulators in autism spectrum disorder: current clinical research. Front Neurosci. (2021) 15:753274. 10.3389/fnins.2021.753274 34916897 PMC8669810

[188] Kavalali ET Monteggia LM. Rapid homeostatic plasticity and neuropsychiatric therapeutics. Neuropsychopharmacol Off Publ Am Coll Neuropsychopharmacol. (2023) 48:54–60. 10.1038/s41386-022-01411-4 35995973 PMC9700859

[189] Winkel F Ryazantseva M Voigt MB Didio G Lilja A Llach Pou M. Pharmacological and optical activation of TrkB in Parvalbumin interneurons regulate intrinsic states to orchestrate cortical plasticity. Mol Psychiatry. (2021) 26:7247–56. 10.1038/s41380-021-01211-0 34321594 PMC8872988

[190] Blackman MP Djukic B Nelson SB Turrigiano GG A. Critical and cell-autonomous role for MeCP2 in synaptic scaling up. J Neurosci. (2012) 32:13529–36. 10.1523/JNEUROSCI.3077-12.2012 23015442 PMC3483036

[191] Fernandes D Santos SD Coutinho E Whitt JL Beltrão N Rondão T. Disrupted AMPA receptor function upon genetic- or antibody-mediated loss of autism-associated CASPR2. Cereb Cortex. (2019) 29:4919–31. 10.1093/cercor/bhz032 30843029 PMC7963114

[192] Sarti F Zhang Z Schroeder J Chen L. Rapid suppression of inhibitory synaptic transmission by retinoic acid. J Neurosci. (2013) 33:11440–50. 10.1523/JNEUROSCI.1710-13.2013 23843516 PMC3724332

[193] Soden ME Chen L. Fragile X protein FMRP is required for homeostatic plasticity and regulation of synaptic strength by retinoic acid. J Neurosci Off J Soc Neurosci. (2010) 30:16910–21. 10.1523/JNEUROSCI.3660-10.2010 21159962 PMC3073636

[194] Tatavarty V Torrado Pacheco A Groves Kuhnle C Lin H Koundinya P. Autism-associated Shank3 Is essential for homeostatic compensation in rodent V1. Neuron. (2020) 106:769–77. 10.1016/j.neuron.2020.02.033 32199104 PMC7331792

[195] Bülow P Murphy TJ Bassell GJ Wenner P. Homeostatic intrinsic plasticity is functionally altered in Fmr1 KO cortical neurons. Cell Rep. (2019) 26:1378–83. 10.1016/j.celrep.2019.01.035 30726724 PMC6443253

[196] Hwang J-Y Monday HR Yan J Gompers A Buxbaum AR Sawicka KJ. CPEB3-dependent increase in GluA2 subunits impairs excitatory transmission onto inhibitory interneurons in a mouse model of fragile X. Cell Rep. (2022) 39:110853. 10.1016/j.celrep.2022.110853 35675768 PMC9671216

[197] Hengen KB Lambo ME Van Hooser SD Katz DB Turrigiano GG. Firing rate homeostasis in visual cortex of freely behaving rodents. Neuron. (2013) 80:335–42. 10.1016/j.neuron.2013.08.038 24139038 PMC3816084

[198] Hengen KB Lambo ME Hooser SDV Katz DB Turrigiano GG. Firing rate homeostasis in visual cortex of freely behaving rodents. Neuron. (2013) 80:335–42. 24139038 10.1016/j.neuron.2013.08.038 PMC3816084

[199] Lambo ME Turrigiano GG. Synaptic and Intrinsic Homeostatic Mechanisms Cooperate to Increase L2/3 Pyramidal Neuron Excitability during a Late Phase of Critical Period Plasticity. J Neurosci Off J Soc Neurosci. (2013) 33:8810–9. 23678123 10.1523/JNEUROSCI.4502-12.2013 PMC3700430

[200] Wu YK Hengen KB Turrigiano GG Gjorgjieva J. Homeostatic mechanisms regulate distinct aspects of cortical circuit dynamics. Proc Natl Acad Sci U S A. (2020) 117:24514–25. 10.1073/pnas.1918368117 32917810 PMC7533694

[201] Gainey MA Aman JW Feldman DE. Rapid disinhibition by adjustment of PV intrinsic excitability during whisker map plasticity in mouse S1. J Neurosci. (2018) 38:4749–61. 10.1523/JNEUROSCI.3628-17.2018 29678876 PMC5956988

[202] Gainey MA Feldman DE. Multiple shared mechanisms for homeostatic plasticity in rodent somatosensory and visual cortex. Philos Trans R Soc Lond B Biol Sci. (2017) 372:20160157. 10.1098/rstb.2016.0157 28093551 PMC5247589

[203] Li L Gainey MA Goldbeck JE Feldman DE. Rapid homeostasis by disinhibition during whisker map plasticity. Proc Natl Acad Sci U S A. (2014) 111:1616–21. 10.1073/pnas.1312455111 24474788 PMC3910567

[204] Garcia-Junco-Clemente P Chow DK Tring E Lazaro MT Trachtenberg JT Golshani P. Overexpression of calcium-activated potassium channels underlies cortical dysfunction in a model of PTEN-associated autism. Proc Natl Acad Sci U S A. (2013) 110:18297–302. 10.1073/pnas.1309207110 24145404 PMC3831440

[205] Ortiz-Cruz CA Marquez EJ Linares-García CI Perera-Murcia GR Ramiro-Cortés Y. Haploinsufficiency of Shank3 increases the orientation selectivity of V1 neurons. Sci Rep. (2022) 12:22230. 10.1038/s41598-022-26402-9 36564435 PMC9789112

[206] Pyronneau A He Q Hwang J-Y Porch M Contractor A Zukin RS. Aberrant Rac1-cofilin signaling mediates defects in dendritic spines, synaptic function, and sensory perception in fragile X syndrome. Sci Signal. (2017) 10:eaan0852. 10.1126/scisignal.aan0852 29114038 PMC5988355

[207] Chang Y-S Owen JP Desai SS Hill SS Arnett AB Harris J. Autism and sensory processing disorders: shared white matter disruption in sensory pathways but divergent connectivity in social-emotional pathways. PLoS ONE. (2014) 9:e103038. 10.1371/journal.pone.0103038 25075609 PMC4116166

[208] Zhou Y Sharma J Ke Q Landman R Yuan J Chen H. Atypical behaviour and connectivity in SHANK3-mutant macaques. Nature. (2019) 570:326–31. 10.1038/s41586-019-1278-0 31189958

[209] Wang W Liu J Shi S Liu T Ma L Ma X. Altered resting-state functional activity in patients with autism spectrum disorder: a quantitative meta-analysis. Front Neurol. (2018) 9:556. 10.3389/fneur.2018.00556 30087648 PMC6066523

[210] Uddin L Supekar K Menon V. Reconceptualizing functional brain connectivity in autism from a developmental perspective. Front Hum Neurosci. (2013) 7: 458. 10.3389/fnhum.2013.00458 23966925 PMC3735986

[211] Pellicano E Burr D. When the world becomes “too real”: a Bayesian explanation of autistic perception. Trends Cogn Sci. (2012) 16:504–10. 10.1016/j.tics.2012.08.009 22959875

[212] Ursino M Serra M Tarasi L Ricci G Magosso E Romei V. Bottom-up vs. top-down connectivity imbalance in individuals with high-autistic traits: an electroencephalographic study. Front Syst Neurosci. (2022) 16:932128. 10.3389/fnsys.2022.932128 36032324 PMC9412751

[213] Buch AM Vértes PE Seidlitz J Kim SH Grosenick L Liston C. Molecular and network-level mechanisms explaining individual differences in autism spectrum disorder. Nat Neurosci. (2023) 26:650–63. 10.1038/s41593-023-01259-x 36894656 PMC11446249

[214] Lefebvre A Tillmann J Cliquet F Amsellem F Maruani A Leblond C. Tackling hypo and hyper sensory processing heterogeneity in autism: From clinical stratification to genetic pathways. Autism Res Off J Int Soc Autism Res. (2023) 16:364–78. 10.1002/aur.2861 36464763

[215] Lombardo MV Lai M-C Baron-Cohen S. Big data approaches to decomposing heterogeneity across the autism spectrum. Mol Psychiatry. (2019) 24:1435–50. 10.1038/s41380-018-0321-0 30617272 PMC6754748

